# Effective detection of staphylococcal infections in human bone tissue using combined raman microscopy and micro-computed tomography

**DOI:** 10.3389/fbioe.2025.1617285

**Published:** 2025-08-21

**Authors:** Richard Andreas Lindtner, Lukas Kampik, Larissa Noack, David Putzer, Rohit Arora, Débora Cristina Coraça-Huber, Michael Schirmer, Gerald Degenhart, Michaela Lackner, Jürgen Popp, Jovan Badzoka, Christoph Kappacher, Christian Wolfgang Huck, Johannes Dominikus Pallua

**Affiliations:** ^1^ Department of Orthopaedics and Traumatology, Medical University of Innsbruck, Innsbruck, Austria; ^2^ Department of Internal Medicine, Clinic II, Medical University of Innsbruck, Innsbruck, Austria; ^3^ Core Facility of MicroCT, University Clinic for Radiology, Medical University of Innsbruck, Innsbruck, Austria; ^4^ Institute for Hygiene and Medical Microbiology, Medical University of Innsbruck, Innsbruck, Austria; ^5^ Leibniz Institute of Photonic Technology, Jena, Germany; ^6^ Institute of Analytical Chemistry and Radiochemistry, University of Innsbruck, Innsbruck, Austria

**Keywords:** bone quality, Raman microscopy, micro-computed tomography, principal component analyses, *Staphylococcus aureus*, *Staphylococcus epidermidis*, bone infection diagnostics, bone graft

## Abstract

Bone infections caused by *Staphylococcus aureus* and *Staphylococcus epidermidis* are serious complications in orthopedic surgery. These infections commonly occur in joint replacements, fracture management, and bone grafting procedures. Rapid and accurate pathogen-specific diagnostic methods are urgently needed to support early clinical decisions. Current culture-based methods are slow and delay effective treatment. This study evaluated the diagnostic value of combining Raman microscopy with high-resolution micro-computed tomography (micro-CT). Human bone samples, either uninfected or inoculated with *S. aureus* or *S. epidermidis*, were analyzed. Raman spectroscopy detected distinct spectral changes in inoculated bones, including reduced intensity of phosphate (v_1_PO_4_^3−^), Amide III, and CH_2_ deformation bands. A single principal component explained 96%–98% of the variance in these infection-related markers. Specifically, the v_1_PO_4_^3−^ and CH_2_ deformation bands effectively differentiated between *S. aureus* and *S. epidermidis* infections, capturing 99%–100% variance. Micro-CT analysis showed significant structural changes in inoculated bones. Trabecular volume, number, and spacing were particularly affected. Among these, VOX-BV/TV and Mean1 best differentiated between *S. aureus* and *S. epidermidis* infections (both p < 0.0001). Support vector machine (SVM) classification repeated stratified k-folg cross-validation accurately detected inoculation status. Combining Raman and micro-CT features yielded moderately improved classification performance in pathogen-specific discrimination. These findings demonstrate that combining molecular (Raman spectroscopy) and structural (micro-CT) methods allows rapid, non-destructive diagnosis of bone infections. This multimodal approach may improve diagnostic precision, supports timely clinical decisions, and ultimately improves patient outcomes in orthopedic and trauma surgery.

## 1 Introduction

Bone infections represent a serious and potentially life-threatening complication in orthopaedic and trauma surgery, particularly in the context of joint replacement, fracture management, and bone grafting procedures (Izakovicova et al., 2019; Metsemakers et al., 2018). These infections are associated with significant morbidity, prolonged hospitalization, and increased healthcare costs and may lead to implant failure, chronic pain, or even limb loss (Patel, 2023; Bezstarosti et al., 2019). The incidence of bone infections has been rising globally, mainly due to the growing number of orthopaedic interventions in ageing populations and patients with comorbidities such as diabetes mellitus or immunosuppression. For example, fracture-related infections (FRIs) currently occur in 1%–2% of all fracture cases, and their prevalence has increased from 8.4 to 10.7 cases per 100,000 inhabitants between 2008 and 2018 (Walter et al., 2021a). Similarly, the rate of periprosthetic joint infections (PJIs) has risen significantly with an incidence between 23.5 and 27.8 per 100,000 inhabitants in Germany (Rupp et al., 2021; Walter et al., 2021b).

Among the causative pathogens, *Staphylococcus* (*S.*) *aureus* and *S. epidermidis* are responsible for most cases (Grieb et al., 2005; DePaula et al., 2005; Lewis et al., 2011). *S. aureus*, including methicillin-resistant strains (MRSA), is highly virulent and more frequently causes severe infections, often invading bone tissue. This can be followed by the formation of biofilms, which protect the bacteria from antibiotics and host immune responses (Vastel et al., 2004). *S. epidermidis*, although part of the normal skin flora, is notorious for its ability to form biofilms on prosthetic materials and implants, leading to chronic, difficult-to-treat infections (Hartman and Tomasz, 1984; Dym and Zeidan, 2017).

Despite the clinical relevance, early and accurate diagnosis of staphylococcal bone infections remains a significant challenge. The microbiological culture of tissue samples is the gold standard for detecting pathogens; however, these methods have limited sensitivity, long turnaround times, and may yield false negatives due to prior antibiotic therapy or low bacterial loads (Perka and Haas, 2011; Eastlund, 2006). Furthermore, conventional imaging modalities such as X-ray, computer tomography (CT), and magnetic resonance tomography (MRI) can reveal structural damage but cannot detect biochemical or molecular changes in bone composition in early infection stages (Lentino, 2003). Advanced imaging, such as positron emission (PET)-CT and single-photon emission computed tomography (SPECT), have been proposed to increase specificity but are costly, not universally available, and involve radiation exposure (Hinsenkamp et al., 2012). Hence, there is a critical need for non-invasive, rapid, and sensitive methods that allow for structural and molecular bone tissue characterization to identify infections, differentiate pathogens, and evaluate the quality of bone grafts before transplantation (Bury et al., 2021).

Recent advances in spectroscopic techniques offer promising new avenues for bone infection diagnostics. Raman spectroscopy is a powerful, label-free, and non-destructive method that enables molecular characterization of biological tissues, including the analysis of bone mineral content and collagen matrix integrity (Lew and Waldvogel, 2004; Chen et al., 2010; Unkila-Kallio et al., 1994). The Raman spectra display distinct bands corresponding to key mineral and matrix components of bone, such as phosphate (PO_4_^3−^), which represents the mineral phase of hydroxyapatite, and carbonate (CO_3_^2−^), often incorporated as a substitute in the apatite lattice. Additionally, proline and hydroxyproline bands reflect the integrity of the collagen matrix, while CH_2_ deformations and lipid-associated bands provide information about organic content. The Amide bands (Amide I and Amide III), linked to the secondary structure of collagen and CH stretching vibrations, further contribute to a comprehensive understanding of bone quality. By measuring the vibrational modes of molecular bonds, Raman spectroscopy can detect subtle biochemical alterations associated with bacterial infection, such as changes in phosphate (ν1PO_4_^3−^), carbonate, Amide I and III bands, reflecting modifications in mineralization and collagen structure (Pineda et al., 2006). So far previous studies have demonstrated the potential of Raman spectroscopy in detecting pathological changes in bone, including osteoporosis and metastatic lesions (Pineda et al., 2006), but its application for the specific detection of bacterial infections in bone remains underexplored.

While Raman microscopy excels in molecular sensitivity, it does not provide spatial information on bone architecture. To address this limitation, micro-computed tomography (micro-CT) offers high-resolution, three-dimensional visualization of bone microstructure, enabling the quantification of trabecular thickness, number, separation, and overall bone volume (Termaat et al., 2005; Schäfer et al., 2008). Micro-CT has been extensively used to assess bone quality in osteoporosis and to evaluate bone regeneration in preclinical models (Lüllmann-Rauch and Paulsen, 2012). However, it cannot reveal biochemical changes associated with bacterial colonization and biofilm formation (Wolff, 1893). Thus, combining Raman microscopy and micro-CT could provide a comprehensive approach, integrating molecular and structural insights to enhance diagnostic accuracy.

Currently, no integrated platform exists for pathogen-specific detection of bone infection through simultaneous analysis of molecular and structural alterations. Moreover, little is known about how *S. aureus* and *S. epidermidis* differentially affect bone composition and architecture in human tissue. Understanding these differences is critical, as these two pathogens employ distinct mechanisms of bone destruction, immune evasion, and biofilm formation (Varma et al., 2016; Rodrigues et al., 2013). Identifying reliable markers for each species could enable more personalized early therapeutic interventions, including tailored antibiotic regimens and optimized surgical strategies.

Therefore, this study aimed to investigate the combined application of Raman microscopy and micro-CT to identify and differentiate *S. aureus* and *S. epidermidis* infections in human bone tissue. Specifically, we sought to (1) identify molecular and structural markers that distinguish infected from non-infected bone, (2) determine pathogen-specific signatures enabling differentiation between *S. aureus* and *S. epidermidis*, and (3) evaluate the potential of this multimodal approach as a diagnostic tool for clinical application. Our results demonstrate that Raman microscopy reveals significant mineral and collagen component alterations in infected bone, while micro-CT identifies corresponding structural deterioration. Integrating both techniques enabled robust discrimination between infected and uninfected bone, and allowed for pathogen-specific differentiation with high statistical significance. This combined approach could significantly enhance the diagnostic process for bone infections, reduce the risk of undetected contamination in bone grafts, and support early detection and treatment of implant-associated infections and osteomyelitis.

## 2 Methods and materials

### 2.1 Sample collection

Human bone samples used in this study were obtained from femoral heads preserved in a certified local biobank. These femoral heads were collected from patients undergoing total hip arthroplasty due to arthrosis or femoral neck fractures. It's important to note that all donors, out of their own volition, provided written informed consent prior to surgery, confirming their voluntary participation and complete understanding of the biobank procedures. A stringent screening protocol is in place to ensure that only bone material meeting therapeutic standards is accepted into the biobank. This is crucial to maintain the quality and integrity of the samples. Samples that do not qualify—due to incomplete donor screening, insufficient documentation, or other exclusion criteria—are designated for research purposes only. Bone samples contaminated with pathogens are strictly excluded from both therapeutic and research use, regardless of scientific relevance. Donor age and sex are not considered exclusion criteria during the selection process. During surgical retrieval, the femoral heads are carefully cooled and irrigated with sterile 0.9% saline to minimise thermal damage during the osteotomy. This is a crucial step to ensure the quality of the bone samples. Upon collection, cartilage and cortical layers are meticulously removed using a surgical bone saw. Spongiosa is then harvested by extracting bone chips measuring 3–5 mm in diameter using a Noviumagus Bone Mill (Spierings Meische Techniek BV, Nijmegen, Netherlands). A total of 120 bone chips, derived from 40 individual donors, were used in this study. All procedures involving human tissue were conducted in strict adherence to the ethical standards outlined in the Declaration of Helsinki and were approved by the institutional ethics committee (EK 1291/2021).

### 2.2 Experimental setup and sample preparation

For this meticulously designed experimental study, 120 bone chips obtained from 40 patients were randomly assigned to three groups: Forty bone samples were inoculated with *S*. *aureus* (ATCC 29213), 40 with *S*. *epidermidis* (ATCC 12228), and the remaining 40 samples were left untreated to serve as controls. The patient cohort included 22 females and 18 males, with age distribution as follows: five patients under 50 years (two females, three males), eight patients aged 50–60 years (six females, two males), nine patients aged 60–70 years (four females, five males), 16 patients aged 70–80 years (nine females, seven males), and two patients over 80 years (one female, one male). The bacterial cultures were incubated in Mueller-Hinton medium at 37°C for 24 h. Following incubation, the bacterial suspension was adjusted to a concentration of 10^6^ CFU/mL, and 200 µL of this diluted solution was transferred into each well of a multi-well plate. Bone samples were then placed individually in the wells, allowing for biofilm formation, and incubated in an orbital shaker (Edmund Bühler GmbH, Bodelshausen, Germany) at 37°C for 48 h. The control samples were not treated with antibiotics before inoculation, as the porous bone matrix could absorb the antibiotic solution, potentially inhibiting the subsequent growth of the selected bacterial strains.

The samples were kept in a humid chamber to simulate bone tissue contamination and facilitate successful biofilm development. After incubation, the remaining bacterial suspension was discarded, and the bone samples were washed with phosphate-buffered saline (PBS). The samples were subsequently dried in an aspirator (3.2 kPa) for 10 min at room temperature before measurement. Extending the drying time to 24 h did not affect spectral quality, confirming that a 10-min drying period was sufficient for analysis.

### 2.3 Raman microscopy

Raman microscopy was carried out in reflectance mode at ambient temperature using the Senterra II microscope (Bruker, Ettlingen, Germany). A 785 nm laser, chosen for its ability to excite Raman-active vibrations without causing sample damage, served as the excitation source, delivering 100 mW at the source and 25 mW of power at the back aperture of a Zeiss EC EPIPLAN 20×/0.4 objective with grating 400. Spectral acquisition covered a range of 3,600 cm^−1^ to 200 cm^−1^, with a spectral resolution of approximately 4 cm^-1^. Before each measurement, the integration time, which determines the duration of signal accumulation, was adjusted near the scanning area to optimize the signal-to-noise ratio while minimizing the risk of sample damage. Each measurement consisted of 100 scans (accumulations) with an integration time (exposure time) of 1 s per scan. To ensure representative sampling, ROIs were arbitrarily selected across the bone surface. Each ROI was mapped using a 6 × 6 grid, resulting in 36 spectral acquisitions with a spatial step size of 5 μm × 5 µm. This spatial sampling oversamples the optical diffraction limit (∼1 µm), allowing for high-resolution mapping of the bone surface.

### 2.4 Raman data processing

Data processing and image reconstruction were performed using OPUS 8.5 software (Bruker), while spectral data analysis was conducted with Unscrambler X 10.5 (AspenTech, Bedford, MA, United States). The preprocessing steps included a 15-point Savitzky-Golay smoothing algorithm, and area normalization techniques. Area normalization was particularly crucial for comparing peak areas across samples, such as I_958_ or amide I, especially in heterogeneous, porous materials like bone, where excitation volume and beam focus variations may influence spectral intensity (Vieira et al., 2019). Peak intensity and area were extracted using a publicly available Microsoft Excel spreadsheet developed by Vieira et al. (2019), which includes baseline correction, peak height identification, and area calculation via both summation and trapezoidal methods. The baseline correction was performed by fitting a linear baseline between two endpoints (x_1_:y_1_ and x_n_:y_n_) of the peak range, which was subtracted from the raw spectrum to obtain accurate peak heights and areas. Two distinct area calculation methods were employed: one based on the partial summation of peak areas and another utilizing total intensity sum calculations. Statistical analysis of spectral parameters was carried out using GraphPad Prism (version 10, San Diego, CA, United States).

The comparison of these parameters between uninoculated bone and bone inoculated with either *S. aureus* or *S. epidermidis* was performed using a one-way ANOVA, complementing the spectroscopic analysis. A two-tailed t-test was applied to further analyze and differentiate between the two pathogen-infected groups. Identifying significant spectral markers provides valuable information for assessing bone quality and enables a scientifically grounded evaluation of possible bacterial colonization. The spectral markers were derived from band intensities (I), band areas (A), and full width at half maximum (FWHM). While peak positions were recorded during spectral acquisition, their variation across experimental groups was negligible in this dataset and did not contribute meaningfully to discrimination. Their diagnostic relevance is based on statistical significance identified via one-way ANOVA and t-tests. Statistical significance was defined as follows: * (p < 0.05), ** (p < 0.01), *** (p < 0.001), and **** (p < 0.0001). Non-evaluable (n.b.) and non-significant (n.s.) results are noted accordingly.

### 2.5 Raman spectral parameters

Several Raman-derived spectral parameters were evaluated to assess bone composition and mineralization. The MMR, calculated as the phosphate-to-amide I ratio (I_958_/I_1656_), reflects bone mineralization quality (McCreadie et al., 2006; Khalid et al., 2018). The IRSF, derived from the 1/FWHM_958_, provides insights into hydroxyapatite crystal size and organization, which influence bone strength (Freeman et al., 2001). The MiniCarb (A_1070_/A_1450_) represents carbonate substitution in the bone mineral lattice, an essential factor in bone remodeling and metabolic turnover (Grunenwald et al., 2014; Morris and Mandair, 2011). The bone phosphate index (BPI, I_1070_/I_577_) provides additional insights into the mineral composition and relative phosphate incorporation (France et al., 2020). Similarly, the apatite-to-phosphate index (API, I_1103_/I_577_) indicates apatite crystallinity, which is linked to bone mechanical properties (France et al., 2020). The carbonate-to-phosphate ratio (C/P, I_1070_/I_958_) is commonly used to evaluate bone mineral composition and potential alterations due to disease (France et al., 2020). These parameters provide valuable biochemical insights into bone structure, mineralization, and collagen content, supporting the differentiation between uninfected and infected bone tissue.

### 2.6 Principal component analyses (PCA) and loading plots

Principal Component Analysis (PCA), or multivariate analysis of principal components, is a statistical approach widely used in diagnostics and clinical decision-making. This is particularly crucial in cases of osteomyelitis, where early diagnosis can be decisive for the course of the disease (Goswami et al., 2018; Beck-Broichsitter et al., 2015). PCA models were constructed using Unscrambler X 10.5. Spectral data were imported and preprocessed using 15-point Savitzky-Golay smoothing and area normalization to enhance spectral quality for analysis. This approach aimed to identify specific wavelength regions that differ significantly between groups and could serve as spectroscopic markers for pathogen identification.

To visualize the contribution of individual variables to the principal components, a loading plot was generated. This graphical representation helps to determine which spectral regions (Raman shifts, in cm^−1^) contribute most to the variance observed in the principal components across the different groups (Backhaus et al., 2016; Kessler, 2011). Spectral regions that lie close to the zero line in the loadings plot indicate similar values among the groups and are thus less suitable for discrimination. In contrast, spectral regions with highly positive or negative correlation coefficients (ranging from −1 to +1) reflect significant differences between groups and are considered relevant for distinguishing infected from non-infected bone tissue (Kessler, 2011).

### 2.7 Micro-computed tomography and data analysis

Scans were obtained with a second-generation HR-pQCT (XtremeCTII, Scanco Medical, Switzerland). Pre-settings of all scans included a resolution of 30,3 µm isovoxels, an integration time of 46 ms, and a voltage and intensity of 68 kV and 1,470 μA, respectively. The Scanco medical software package was used for direct post-processing, containing VMS (multiprocessing virtual memory-based operating system, ©Hewlett-Packard, Palo Alto United States) and image processing language IPL (Image Processing Language, Scanco Medical AG, Bruttisellen, Switzerland). Image reconstruction was performed using cone-beam back-projection algorithms integrated into the Scanco µCT system (Scanco Medical AG, Brüttisellen, Switzerland). This approach compensates for distortions inherent in cone-beam imaging, ensuring high-resolution reconstructions of the scanned bone samples. The workstation operated with the Image Processing Language (IPL) software (Scanco Medical AG, Switzerland), enabling precise alignment of datasets, segmentation to differentiate structural components, and three-dimensional reconstruction for volumetric analysis. Following Scanco Medical’s standardized contouring and morphing guidelines, a semi-automated segmentation process was applied to accurately distinguish trabecular and cortical bone, ensuring reliable differentiation between the metabolically active trabecular network and the dense cortical layer. Post-processing was conducted using IPL software, where key structural and morphometric parameters were extracted to assess bone microarchitecture. Total bone volume was determined using VOX-TV, representing the entire volume of the region of interest, while VOX-BV quantified the portion of this volume occupied by mineralized bone tissue. The ratio of these two parameters, VOX-BV/TV, provided an essential measure of overall bone density. Conn-Dens was calculated to evaluate the connectivity of the trabecular network, describing the number of connected trabecular structures per unit volume. The TRI-SMI was used to characterize the plate-rod structure of trabeculae, with lower values indicating a more plate-like structure, which is typically associated with more excellent mechanical stability. Detailed trabecular architecture was analyzed through DT-Tb.N, reflecting the density of trabeculae per unit length and DT-Tb.Th provided an average measurement of the trabecular strut thickness. DT-Tb.Sp quantified the average distance between trabeculae, indicating bone porosity. The variability within these parameters was assessed using their respective standard deviations: DT-Tb.(1/N). SD, DT-Tb.Th.SD, and DT-Tb.Sp.SD, ensuring a more comprehensive assessment of trabecular heterogeneity. Additionally, Mean1 and Mean2 were derived as mean grayscale intensity values, providing an estimate of tissue mineral density and variations in bone mineralization. These combined parameters offer a detailed evaluation of bone microarchitecture, structural integrity, and mineral distribution, which are essential for understanding the biomechanical properties of bone under different experimental conditions (Ostertag et al., 2014; Cooper et al., 2007).

### 2.8 Statistical analysis of micro-CT data

Descriptive statistics, including mean, median, and standard deviation, were computed for each group of bone samples (uninoculated, *S*. *aureus*-inoculated, and *S*. *epidermidis*-inoculated). One-way ANOVA assessed significant differences in trabecular bone parameters, focusing on trabecular number, thickness, separation, connectivity density, and structural heterogeneity. This analysis revealed significant differences, with statistical significance set at P < 0.05. Two-way ANOVA was applied to investigate the interaction between infection status and trabecular bone properties. All statistical analyses were performed using the advanced GraphPad Prism (version 10, San Diego, CA, USA), a state-of-the-art statistical software. The 3D visualizations of reconstructed bone microarchitecture were generated using the cutting-edge IPL-based 3D rendering tools from Scanco Medical AG, demonstrating the use of the latest technology in the research.

### 2.9 Classification of bone inoculation status

To prepare the dataset for supervised classification, Raman spectroscopy markers and micro-CT parameters were merged into a unified feature matrix. For Raman spectroscopy markers, three measurements were acquired per bone sample at different locations. To prevent pseudoreplication and information leakage, Raman indices were averaged per sample across the three measurement points, resulting in a single Raman feature vector per sample. Micro-CT parameters were already available on a per-sample basis. To ensure completeness of the combined dataset, missing values were imputed using the class-specific median of the respective variable. This resulted in a final dataset comprising 39 uninfected bone samples, 40 samples inoculated with *S. aureus*, and 40 samples inoculated with *S. epidermidis*, each with a full set of Raman and micro-CT features. Categorical labels were binarized to perform two binary classification tasks:

1. inoculated (*S. aureus + S. epidermidis*) vs. uninfected, and
2. *S. aureus* vs. *S. epidermidis*

All machine learning analysis and statistical evaluations were implemented in Python (v.3.10) using the scikit-leran and SciPy libraries. Additionally, PCA was performed in MATLAB (MATLAB Version: 24.1.0.2628055 (R2024a) Update 4, Natick, Massachusetts: The MathWorks Inc.) for exploratory feature analysis, enabling identification of the most influential Raman markers and micro-CT parameters associated with inoculation status and pathogen type.

Classification performance was assessed using a linear Support Vector Machine (SVM) combined with feature standardization. Evaluation was conducted via repeated stratified k-fold cross-validation (5 folds, 10 repeats), yielading 50 iterations per classification task. This approach provided stable performance estimates across balanced splits while accounting for variability due to limited sample size.

Multiple performance metrics (accuracy, precision, recall, and F1-score) were reported to provide a comprehensive evaluation of classifier performance. Statistical comparisons were restricted to accuracy, which served as the primary evaluation metric. This approach avoids inflation of the type I error rate associated with multiple hyptoheses testing, while still offering insight into secondary model characteristics.

Statistical comparison between models focused on accuracy as primary metric. For each classification task – infection subtype (*S. aureus* vs. S. epidermidis) and infection detection (inoculated vs. unioculated) – Wilcoxon signed-rank tests (two-sided) were applied to compare the combined model (Raman + microCT) against the respective unimodal baselines (Raman only, CT only). Tests were performed over the 50 accuracy values obtained from cross-validation. As only two pairwise comparisons were conducted per task, no correction for multiple testing was applied.

## 3 Results

In this experimental study, 120 human bone samples were analyzed using Raman microscopy and micro-CT to differentiate between infected and non-infected bone samples and subsequently to distinguish between the most common pathogens of bone infection, *S. aureus* and *S. epidermidis*.

### 3.1 Averaged Raman spectra of human bone samples with and without bacterial inoculation

**FIGURE 1:**
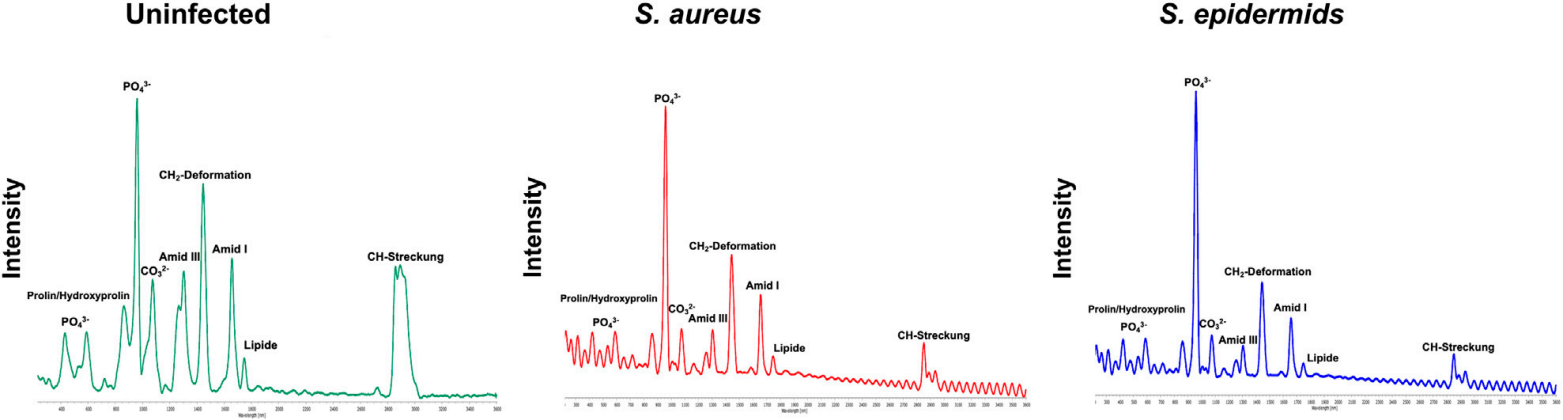
Averaged Raman spectra of uninfected bone (top), bone inoculated with *S. aureus* (middle), and bone inoculated with *S. epidermidis* (bottom). Key spectral markers are labelled, including phosphate (PO_4_^3−^), proline/hydroxyproline, carbonate (CO_3_^2−^), Amide I, Amide III, CH_2_ deformations, lipids, and CH stretching bands. The spectra show pathogen-induced reductions in Amide bands, lipid signals, and CH stretching in infected samples, indicating degradation of the organic bone matrix. Additionally, differences between *S. aureus* and *S. epidermidis* suggest varying pathogen-specific impacts on bone composition.

The detailed analysis of the Raman spectra reveals substantial molecular changes in bone tissue following bacterial colonization. The mean Raman spectra are shown in Figure 1. The comparison between an uninoculated bone and a bone sample inoculated with *S. aureus* shows differences in the analyzed vibrational modes and spectral ranges. A pronounced decrease in the phosphate v2 bending mode is particularly striking, typically found between 420 and 450 cm^−1^ in the spectral range. This reduction indicates potential alterations in the phosphate structure of bone induced by bacterial infection. In contrast, the phosphate ν4 bending mode, located between 587 and 604 cm^−1^, remains unaffected mainly, showing only minimal changes, which suggests that this specific vibrational mode is less influenced by bacterial activity. The v_1_ symmetric stretching mode of phosphate, found at around 960–961 cm^−1^, also shows a slight decrease in intensity; however, this reduction is less markable than the v_2_ mode. Particularly prominent are the alterations observed in the region corresponding to type A and type B carbonate groups, between 1,070 and 1,103 cm^−1^. Here, a significant decrease in peak intensity indicates substantial modifications of carbonate groups within the bone matrix.

In addition to these mineral components, a marked reduction in Amide III intensity is observed, reflecting the degradation of the collagen matrix. Similarly, peaks associated with CH_2_ deformations, the Amide I band at 1,656 cm^−1^, and lipid-related signals are considerably reduced in infected samples. Compared to non-infected controls, these bands appear markedly less intense in samples infected with *S. aureus*. However, the most pronounced reduction is found in the biphasic peak of the CH stretching region, further supporting the notion that bacterial colonization profoundly affects both organic and inorganic components of bone, including proteins and lipids, thereby significantly lowering their Raman spectral intensities.

In summary, Raman spectra of bone samples infected with either *S. aureus* or *S. epidermidis* display similar significant reductions in phosphate, carbonate, protein, and lipid bands. Nevertheless, *S. epidermidis* appears to cause more severe alterations in certain spectral regions, particularly within the CH stretching domain, than *S. aureus*. Further statistical analysis of the Raman spectral parameters was warranted to validate and quantify the spectral observations and identify diagnostically relevant markers.

### 3.2 Evaluation of Raman spectral band ratios for bone tissue analysis

**FIGURE 2:**
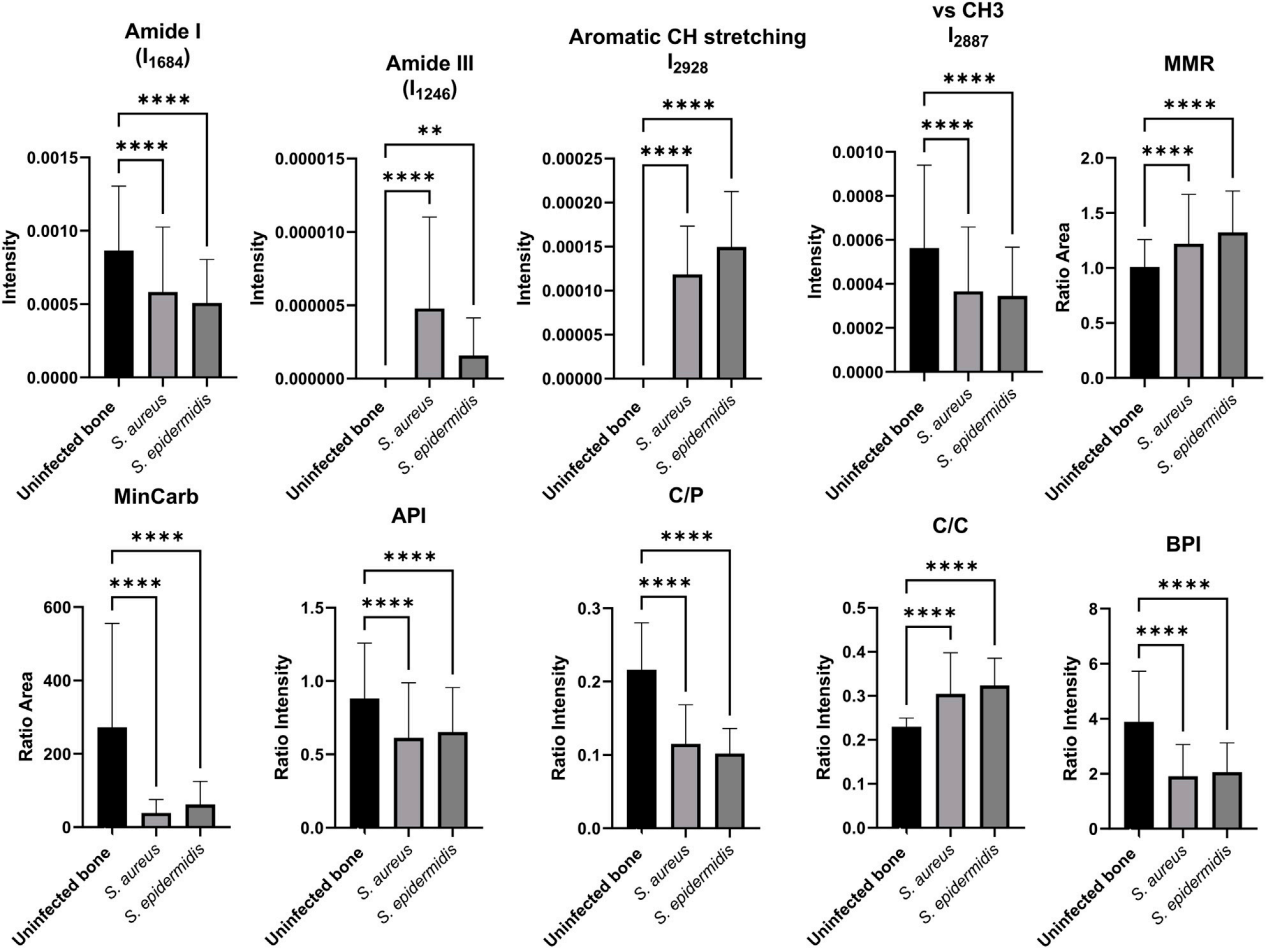
Comparison of key Raman spectroscopic markers characterizing human bone tissue in uninfected and inoculated samples. The bar plots display the relative intensities and ratios of essential Raman bands, including phosphate (958 cm^−1^), Amide I (1,656 cm^−1^), Amide III (1,246 cm^−1^), CH stretching bands (2,929 cm^−1^, 2,940 cm^-1^), and carbonate (1,070 cm^−1^), as well as diagnostic ratios: MMR, MinCarb, API, C/P, C/C, and BPI. Data are shown as mean ± SD; * (p < 0.05), ** (p < 0.01), *** (p < 0.001), **** (p < 0.0001) indicate levels of significance based on one-way ANOVA and t-tests.

An Excel tool calculated intensities and peak areas of characteristic Raman bands based on the acquired Raman microscopic data (Vieira et al., 2019). The results of the one-way ANOVA are visualized in Figure 2 and reveal several bone-specific markers with significant differences when comparing infected to uninoculated bone (p < 0.0001). Notably, a substantial decrease in the Amide I (1,656 cm^−1^) and Amide III (1,246 cm^−1^) bands is observed in both *S. aureus* and *S. epidermidis*-infected samples. These Amide bands are directly associated with collagen integrity, reflecting the secondary protein structure of the organic matrix. Their significant reduction (****p < 0.0001) suggests substantial degradation of collagen fibres in inoculated bone.

Additionally, CH stretching bands (2,929 cm^−1^ and 2,940 cm^−1^), which are indicative of aliphatic and methyl (CH_3_) groups in lipids and proteins, show markedly reduced intensities in infected bone, pointing to a loss of organic content, including collagen and lipids. Interestingly, the CH stretching bands are even more significantly reduced in *S. epidermidis*-inoculated bone than *S. aureus*, indicating potentially more aggressive degradation of the organic matrix by *S. epidermidis*. The Mineral/Matrix Ratio (MMR), typically calculated as phosphate to Amide I (A_958_/A_1656_), show statistically significant changes between infected and uninfected bone, indicating that this specific ratio may be a robust marker for distinguishing infected from uninfected bone. It should be noted that mineral-to-matrix ratios such as A_958_/A_1656_ can also be influenced by underlying bone pathologies. For example, osteoarthritis (OA) may increase MMR locally due to subchondral bone sclerosis and increased mineral deposition (Boskey and Imbert, 2017). In contrast, osteoporosis (OP) is often associated with reduced mineral and collagen content, leading to lower MMR values and increased fracture risk (Recker et al., 2009). Raman-based MMR has been proposed as a discriminative marker across such bone conditions (Mandair and Morris, 2015). While infection-induced changes dominate in our dataset, baseline variability due to comorbid bone disease should be considered in clinical translation. The mineral carbonate content (MinCarb, A_1070_/A_1450_) was significantly reduced in both infection groups, reflecting a substantial loss of carbonate substitutions within the bone apatite lattice, which is critical for bone quality and stability. Moreover, several structural and compositional ratios highlighted clear mineral quality and composition differences. The apatite-to-phosphate ratio (API, I_1103_/I_577_) and carbonate-to-phosphate ratio (C/P, I1070/I958), both critical indicators of mineral crystallinity and carbonate substitution, were significantly reduced in inoculated bones, suggesting bacterial-mediated demineralization and altered crystal structure. These findings are further supported by the carbonate-to-carbonate ratio (C/C, I1103/I1070), which reflects changes in carbonate incorporation within the apatite lattice. This ratio also showed highly significant differences between infected and non-inoculated bone samples. Interestingly, the Bone Phosphate Index (BPI, I_1070_/I_577_), a marker associated with the balance between carbonate and phosphate in bone minerals, was also significantly reduced, indicating a disruption of normal mineral substitution patterns due to bacterial activity.

### 3.3 Diagnostic performance of principal component analysis (PCA)

Given that the diagnostic value of PCA applied to Raman spectral data has already been demonstrated in numerous studies, this method was also employed in the present experimental study to distinguish between uninfected and inoculated bone tissue samples (Pallua et al., 2012; Pallua et al., 2017; Mozhaeva et al., 2022). The basis for PCA was the averaged Raman spectra of the 120 analyzed bone samples.

**FIGURE 3:**
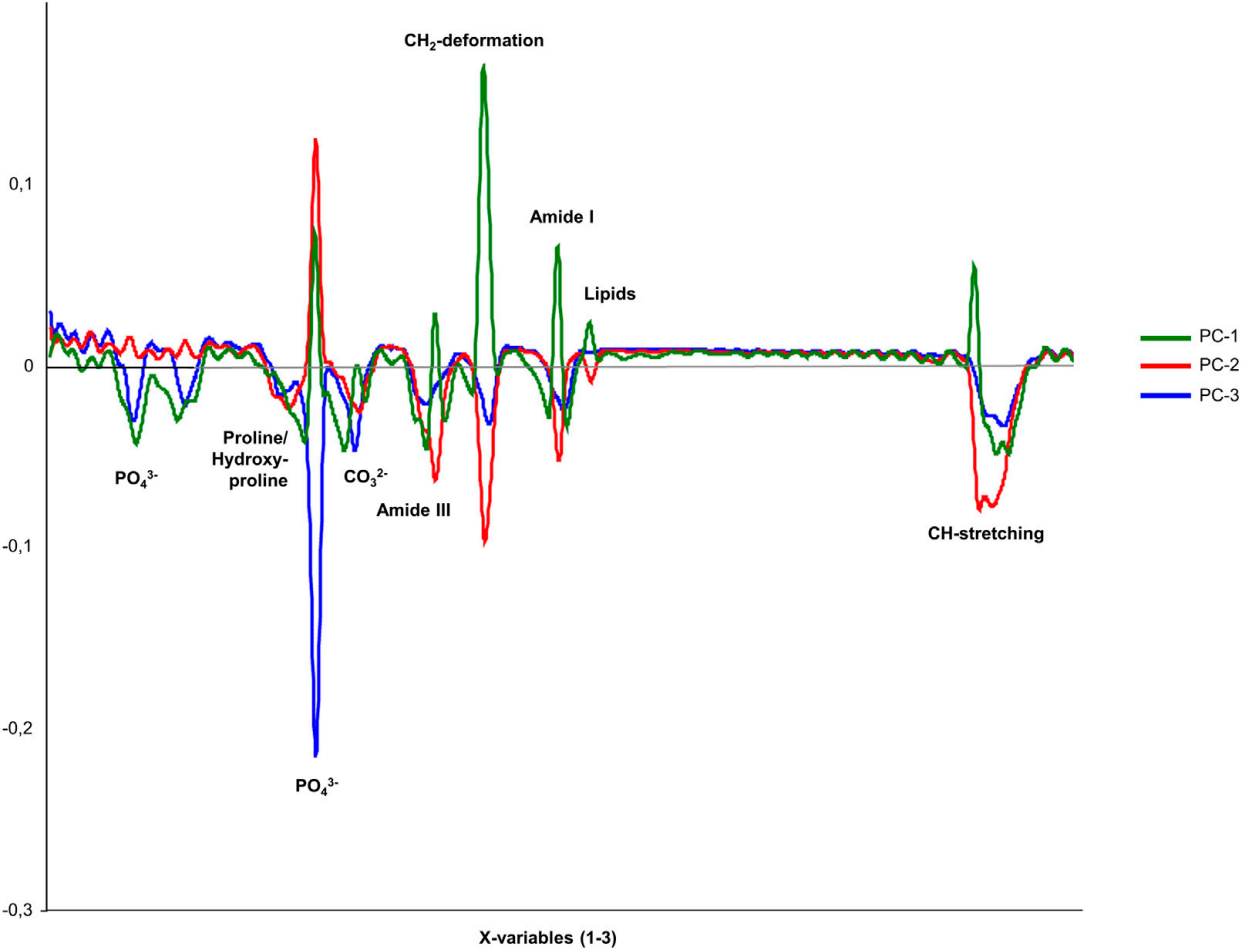
Loadings plot for the three principal components PC-1 (unincolated bone), PC-2 (*S. aureus*), and PC-3 (*S. epidermidis*), showing the correlation coefficients of individual variables within the spectral range of 200–3,000 cm^−1^ for comparing inoculated and uninfected bone.

The loadings plots of all three groups, presented in Figure 3, demonstrate that the correlation coefficients vary between the bone samples in specific spectral regions. This observation confirms the existence of distinct spectral parameters suitable for differentiating between inoculated and uninfected bone tissue. In particular, the spectral regions associated with ν1PO_4_^3-^, Amide III, Amide I, CH_2_ deformations, and CH stretching bands show strong potential for group discrimination.

**FIGURE 4:**
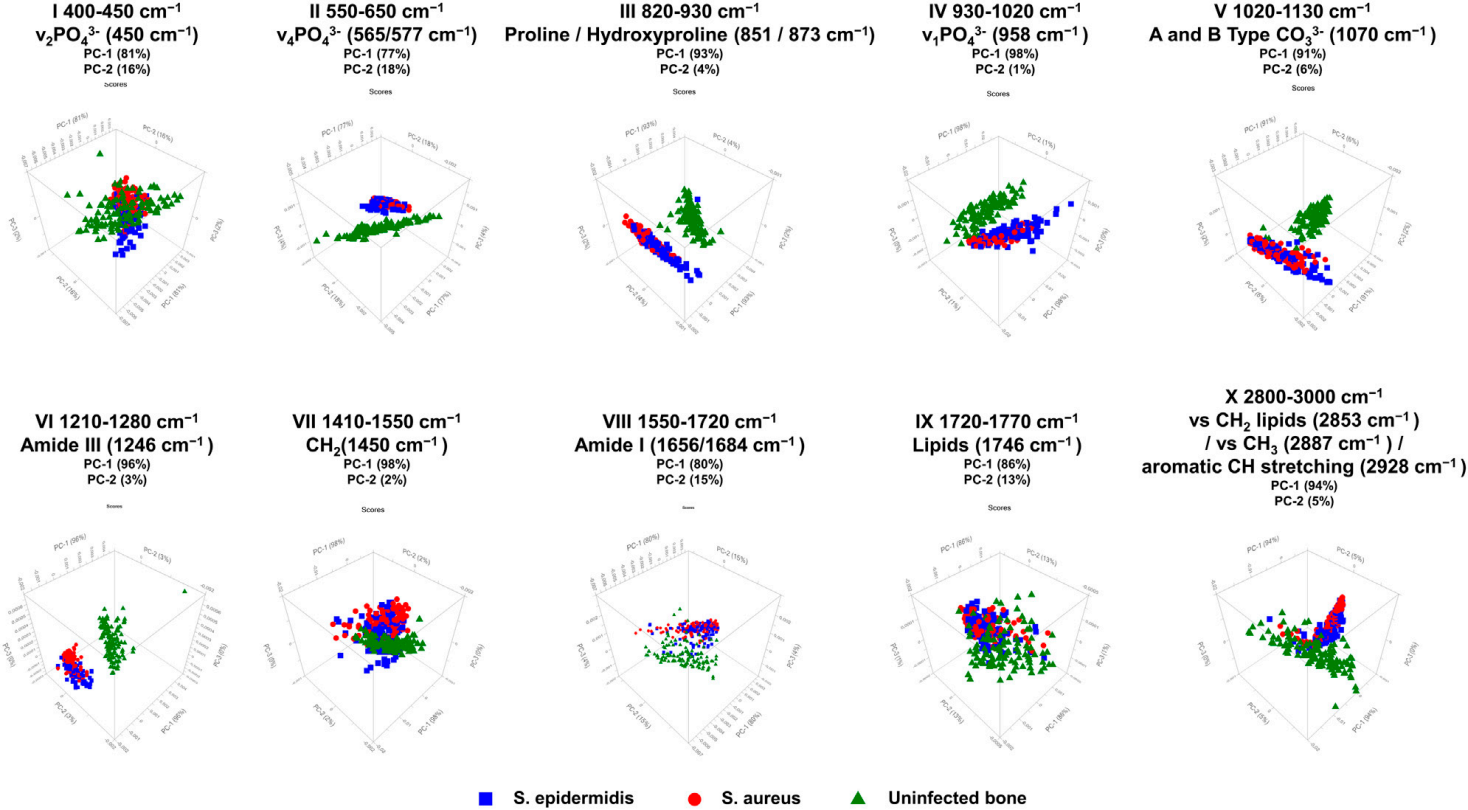
PCA 3D score plots for ten selected spectral regions of Raman measurements in human bone tissue. The figure shows score plots of PC-1 vs. PC-2 vs. PC-3, derived from Raman spectral data obtained with the SENTERRA II Raman microscope. The PCA was performed to distinguish between non-infected (green triangles), *S. aureus*-infected (red circles), and *S. epidermidis*-infected (blue squares) bone samples. A total of ten characteristic spectral regions were analyzed as potential discriminatory markers. The PCA score plots indicate that specific spectral areas, particularly those associated with phosphate (v_1_PO_4_^3−^, v_2_PO_4_^3−^), carbonate (CO_3_^2−^), collagen-related Amide bands (Amide I, III), and CH stretching, allow for differentiation between inoculated and uninfected bone, as well as partial discrimination between *S. aureus* and *S. epidermidis* infections. The percentage of variance explained by each principal component (PC-1 and PC-2) is indicated for each spectral region, highlighting the dominant contribution of PC-1 to group separation.

Figure 4; Table 1 display the results of the PCA analysis, further examining ten spectral regions as potential discriminatory markers for bone tissue. As shown in Figure 4; Table 1, the performed PCA results confirm and support the optical analysis findings based on the loadings plots and the statistical results. PCA typically focuses on the first two principal components, PC-1 and PC-2, since these components account for approximately 95% of the relevant variance in the dataset. All analyzed spectral regions allow a clear separation of bone samples based on PC-1 and PC-2, reaching or exceeding a combined explained variance of 95%. Particularly relevant are regions IV, VI, and VII, corresponding to v_1_PO_4_^3-^ (region IV), Amide III (region VI), and CH_2_ deformations (region VII). In these specific spectral regions, PC-1 alone accounts for 98%, 96%, and 98%, respectively, which highlights that discrimination between inoculated and uninfected bone can be achieved with a single principal component based on these molecular markers. Thus, PCA confirms that all analyzed spectral regions are suitable for distinguishing infected from uninfected bone tissue, with v_1_PO_4_^3−^ (930–1,020 cm^−1^), Amide III (1,210–1,280 cm^−1^), and CH_2_ deformations (1,410–1,550 cm^−1^) standing out as the most significant discriminatory markers.

**TABLE 1:** PCA of uninfected bone samples and bone samples inoculated with either *S. aureus* or *S. epidermidis*, measured with SENTERRA II.

Wave number Range number	PCA	Assignment	Spectral region
I	PC-1 (81%) PC-2 (16%)	v_2_PO_4_^3−^ (450 cm^−1^)	400 to 450 cm^-1^
II	PC-1 (77%) PC-2 (18%)	v_4_PO_4_^3-^ (565/577 cm^-1^)	550 to 650 cm^-1^
III	PC-1 (93%) PC-2 (4%)	Proline/Hydroxyproline (851/873 cm^-1^)	820 to 930 cm^-1^
IV	PC-1 (98%) PC-2 (1%)	v_1_PO_4_^3-^ (958 cm^-1^)	930 to 1,020 cm^-1^
V	PC-1 (91%) PC-2 (6%)	Typ-A und -B Carbonat (1,070 cm^-1^)	1,020 to 1,130 cm^-1^
VI	PC-1 (96%) PC-2 (3%)	Amide III (1,246 cm^-1^)	1,210 to 1,280 cm^-1^
VII	PC-1 (98%) PC-2 (2%)	CH_2_ (1,450 cm^-1^)	1,410 to 1,550 cm^-1^
VIII	PC-1 (80%) PC-2 (15%)	Amide I (1,656/1,684 cm^-1^)	1,550 to 1720 cm^-1^
IX	PC-1 (86%) PC-2 (13%)	Lipids (1746 cm^-1^)	1720 to 1770 cm^-1^
X	PC-1 (94%) PC-2 (5%)	CH_2_-Lipids (2,853 cm^-1^)/CH_3_ (2,887 cm^-1^)/CH-stretching (2,928 cm^-1^)	2,800 to 3,000 cm^-1^

**FIGURE 5:**
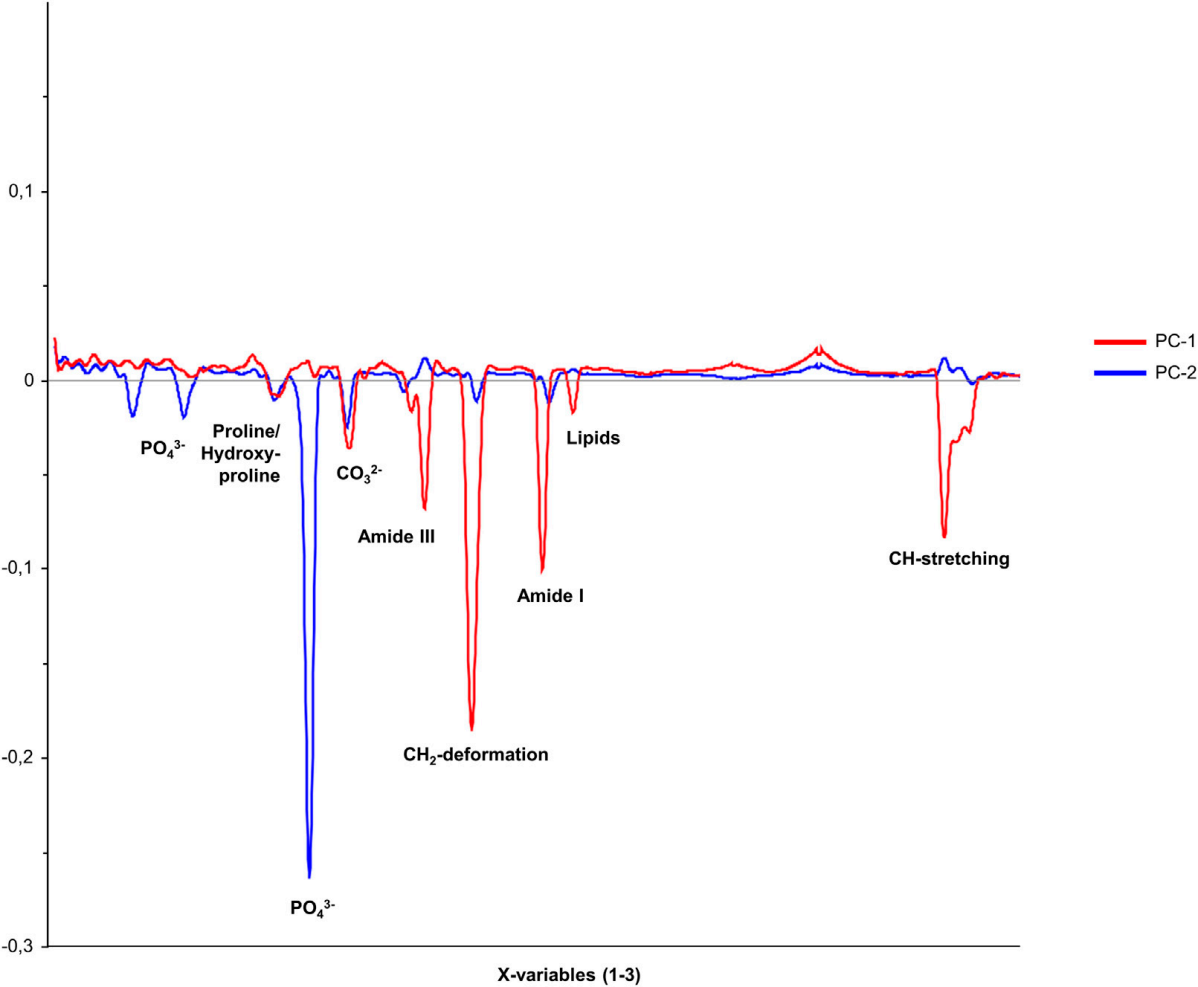
Loadings plot for the two principal components, PC-1 and PC-2, showing the correlation coefficients of individual variables within the spectral range of 200–3,000 cm^−1^ for the comparison between bone samples inoculated with *S. aureus* and *S. epidermidis*.

**FIGURE 6:**
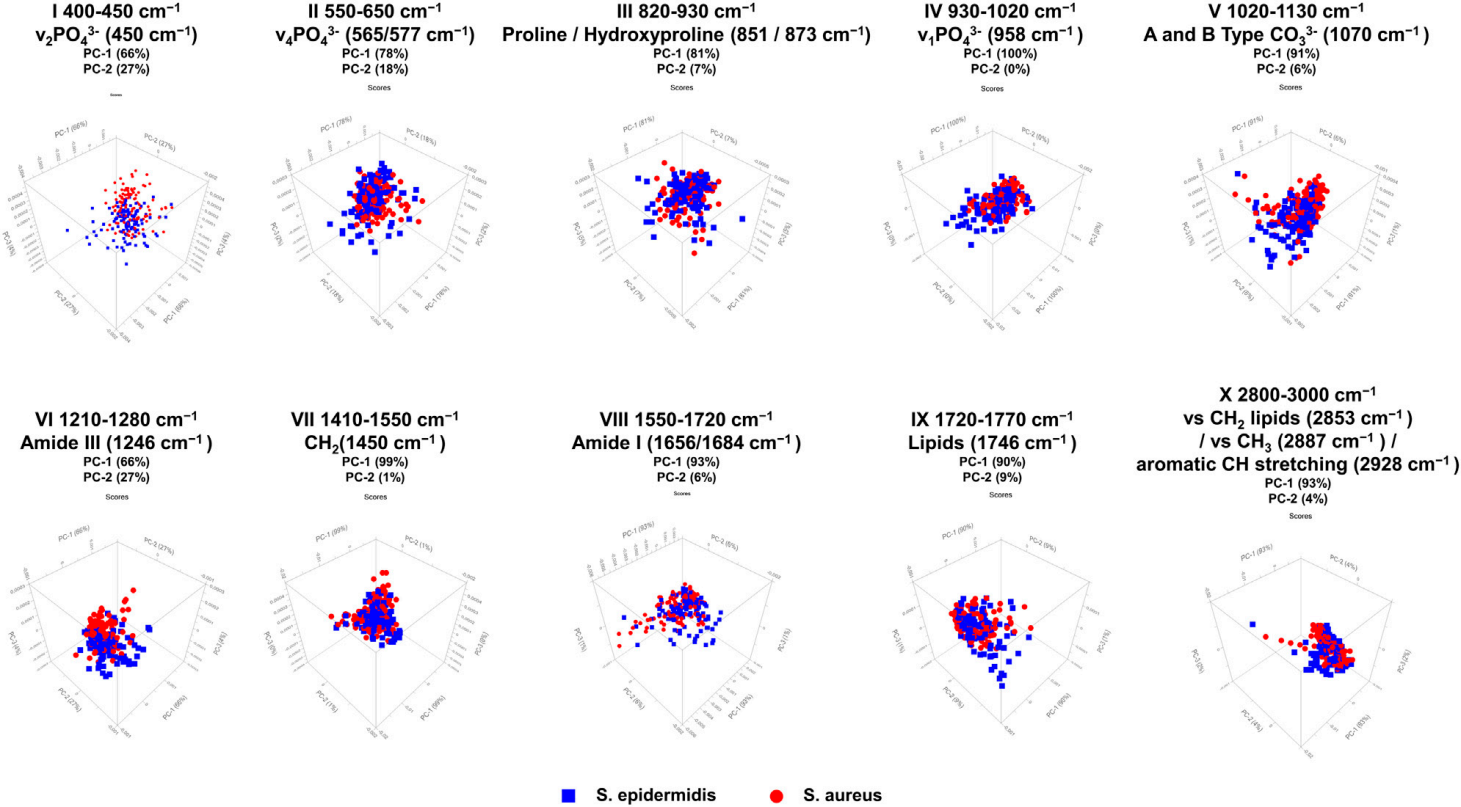
PCA 3D score plots for ten selected Raman spectral regions comparing bone samples infected with *S. aureus* (red circles) and *S. epidermidis* (blue squares). PCA was performed on Raman spectral data acquired with the SENTERRA II Raman microscope to assess pathogen-specific differences in infected bone tissue. The score plots display the separation based on PC-1 and PC-2, which explain the majority of spectral variance in each region, with PC-1 generally accounting for the dominant proportion of variance. The results demonstrate that specific spectral areas, particularly phosphate (v_1_PO_4_^3−^, v_2_PO_4_^3−^), Amide III, CH_2_ deformations, and CH stretching bands, are highly relevant for discriminating between *S. aureus* and *S. epidermidis*-inoculated bone samples.

In the next step, a loadings plot was again used to visualize the correlations of individual variables, including the averaged spectra of bone samples inoculated with *S. aureus* and *S. epidermidis*. As shown in Figure 5, the spectral regions corresponding to PO_4_^3−^, proline/hydroxyproline, CO_3_^2−^, Amide III, CH_2_ deformation, Amide I, lipids, and CH stretching showed negative correlation coefficients suggesting that the variance between the inoculated bone samples is found within these spectral regions and that PO_4_^3-^, Amide III, CH_2_ deformation, Amide I, and CH stretching allow for meaningful differentiation in the optical analysis. For this purpose, an additional PCA was conducted with the 40 samples inoculated with *S. aureus* and 40 samples inoculated with *S. epidermidis* (results presented in Figure 6; Table 2).

**TABLE 2:** PCA of bone samples infected with *S. aureus* and *S. epidermidis* measured using the SENTERRA II Raman microscope.

Wave number Range number	PCA	Assignment	Spectral region
I	PC-1 (66%) PC-2 (27%)	v_2_PO_4_^3-^ (450 cm^-1^)	400 to 450 cm^-1^
II	PC-1 (78%) PC-2 (18%)	v_4_PO_4_^3-^ (565/577 cm^-1^)	550 to 650 cm^-1^
III	PC-1 (81%) PC-2 (7%)	Proline/Hydroxyproline (851/873 cm^-1^)	820 to 930 cm^-1^
IV	PC-1 (100%) PC-2 (0%)	v_1_PO_4_^3-^ (958 cm^-1^)	930 to 1,020 cm^-1^
V	PC-1 (91%) PC-2 (6%)	Typ-A und -B Carbonat (1,070 cm^-1^)	1,020 to 1,130 cm^-1^
VI	PC-1 (66%) PC-2 (27%)	Amide III (1,246 cm^-1^)	1,210 to 1,280 cm^-1^
VII	PC-1 (99%) PC-2 (1%)	CH_2_ (1,450 cm^-1^)	1,410 to 1,550 cm^-1^
VIII	PC-1 (93%) PC-2 (6%)	Amide I (1,656/1,684 cm^-1^)	1,550 to 1720 cm^-1^
IX	PC-1 (90%) PC-2 (9%)	Lipide (1746 cm^-1^)	1720 to 1770 cm^-1^
X	PC-1 (93%) PC-2 (4%)	CH_2_-Lipids (2,853 cm^-1^)/CH_3_ (2,887 cm^-1^)/CH-stretching (2,928 cm^-1^)	2,800 to 3,000 cm^-1^

When comparing bone samples infected with S. aureus and S. epidermidis, PCA was used — just as in the analysis of infected versus non-infected samples — to identify spectral regions that enable pathogen-specific differentiation. Considering the first two principal components, the spectral regions II (v_4_PO_4_^3−^ in the range of 550–650 cm^−1^), IV (v_1_PO_4_^3−^ between 930 and 1,020 cm^−1^), V (type A and B carbonate between 1,020 and 1,130 cm^−1^), VII (CH_2_ deformations within 1,410–1,550 cm^−1^), VIII (Amide I between 1,550 and 1720 cm^−1^), IX (lipids in the range of 1720–1770 cm^−1^), and X (CH_2_ lipids/CH_3_/CH stretching between 2,800 and 3,000 cm^−1^) are particularly relevant for pathogen-specific assignment. These spectral regions (II, IV, V, VII, VIII, IX, and X) reach a combined variance explanation of 95% through PC-1 and PC-2, indicating that the bone samples can be reliably differentiated within these spectral ranges using these principal components. Regions IV and VII stand out as the most meaningful Raman bands. Both phosphate v_1_PO_4_^3−^ (930–1,020 cm^−1^) and CH_2_ deformations (1,410–1,550 cm^−1^) can be distinguished almost entirely through PC-1 alone, accounting for 100% and 99% of the variance, respectively, making these spectral regions highly suitable markers for differentiating between the two pathogens and providing clear and robust results.

### 3.4 Comparison of micro-CT parameters between infected and non-infected human bone samples

**FIGURE 7:**
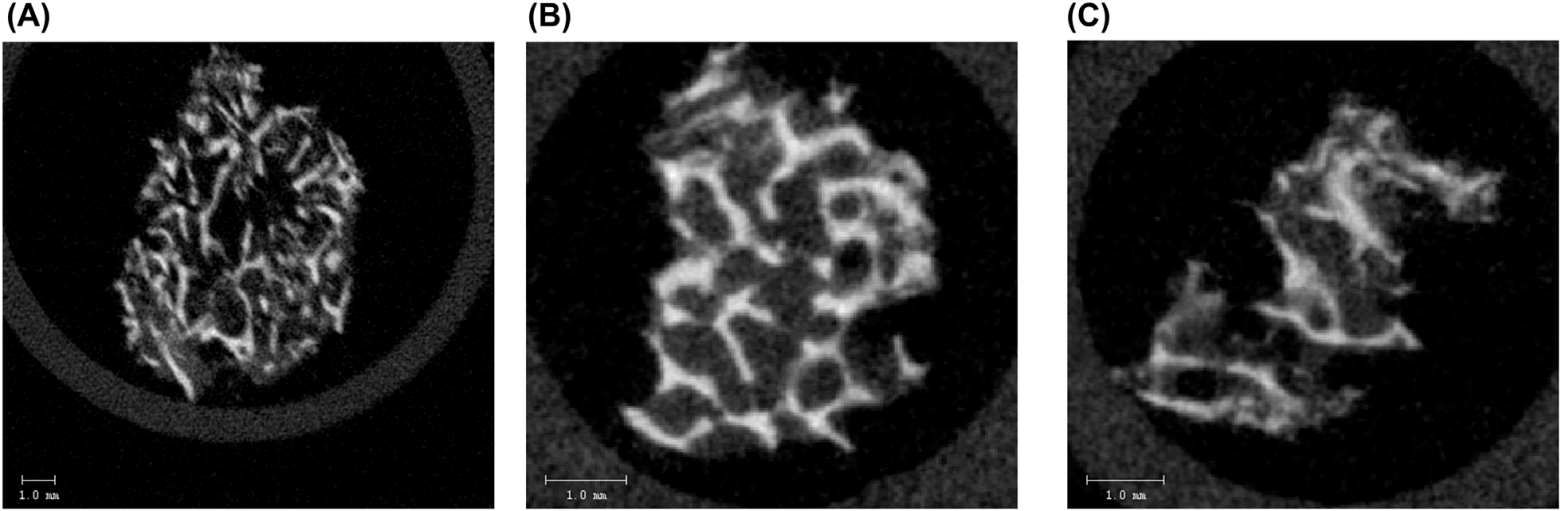
Representative micro-CT cross-sectional images of a human **(A)** non-infected bone sample, showing intact and dense trabecular structure, **(B)** bone sample infected with *S. aureus*, showing disrupted and thinned trabeculae with reduced structural integrity, and **(C)** bone sample infected with *S. epidermidis*, exhibiting pronounced structural degradation and loss of trabecular connectivity. The scale bar represents 1.0 mm. These images highlight the pathogen-induced structural alterations in the trabecular bone as assessed by micro-CT analysis.

**FIGURE 8:**
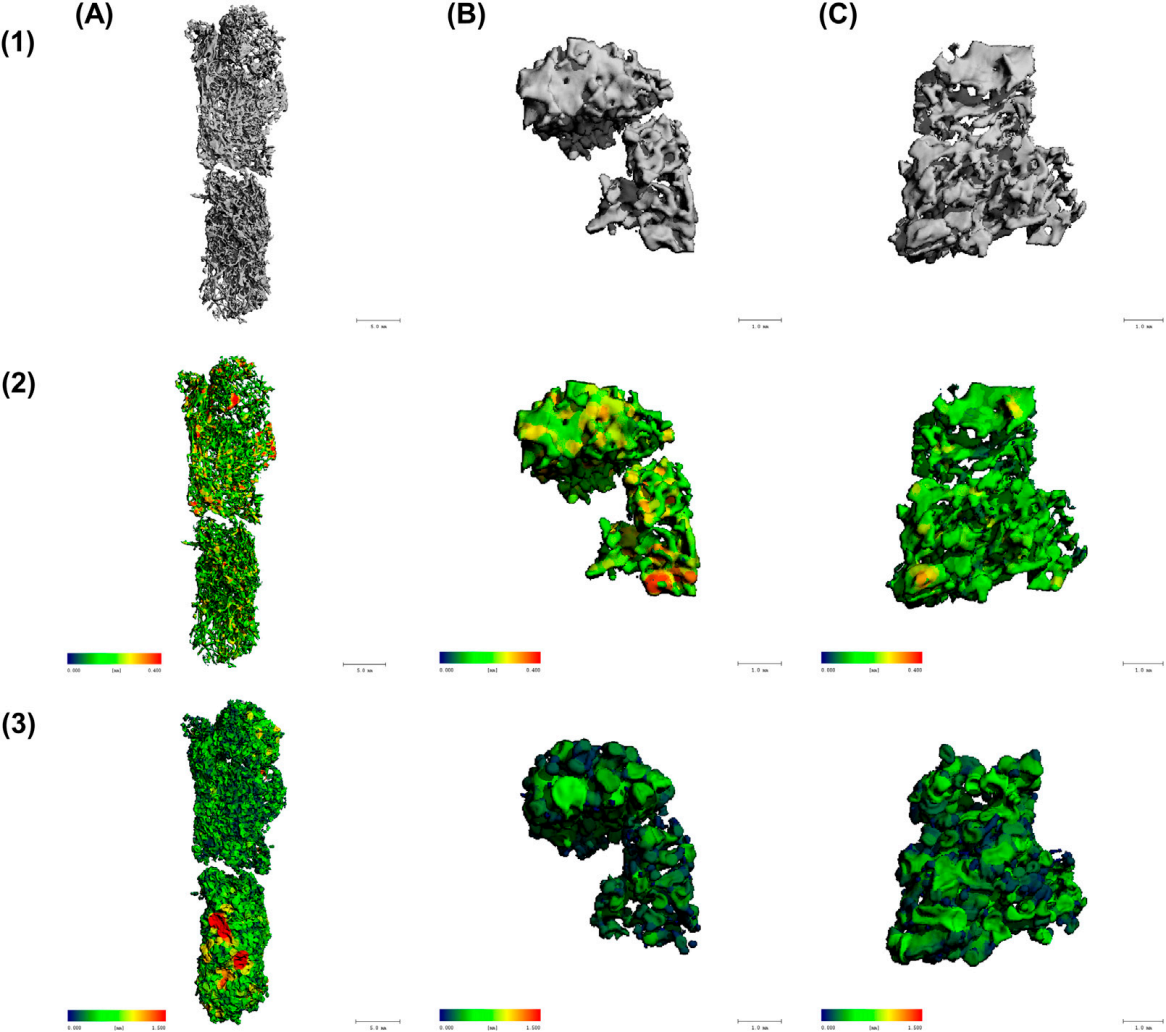
Micro-CT analysis of trabecular bone structure in **(A)** non-infected bone, **(B)** bone inoculated with *S. aureus*, and **(C)** bone inoculated with *S. epidermidis*. Different structural parameters are displayed in the three lines with (1) Trabecular structure in 3D segmentation, showing overall morphology, (2) Trabecular thickness (DT-Tb.Th), with red/yellow regions indicating higher trabecular thickness (up to 0.4 mm) and blue/green regions representing lower trabecular thickness (close to 0 mm), and (3) Trabecular separation (trabecular separation) analysis, with red/yellow areas signifying higher trabecular separation (up to 1.5 mm) and blue/green areas denoting lower trabecular separation (close to 0 mm).

**FIGURE 9:**
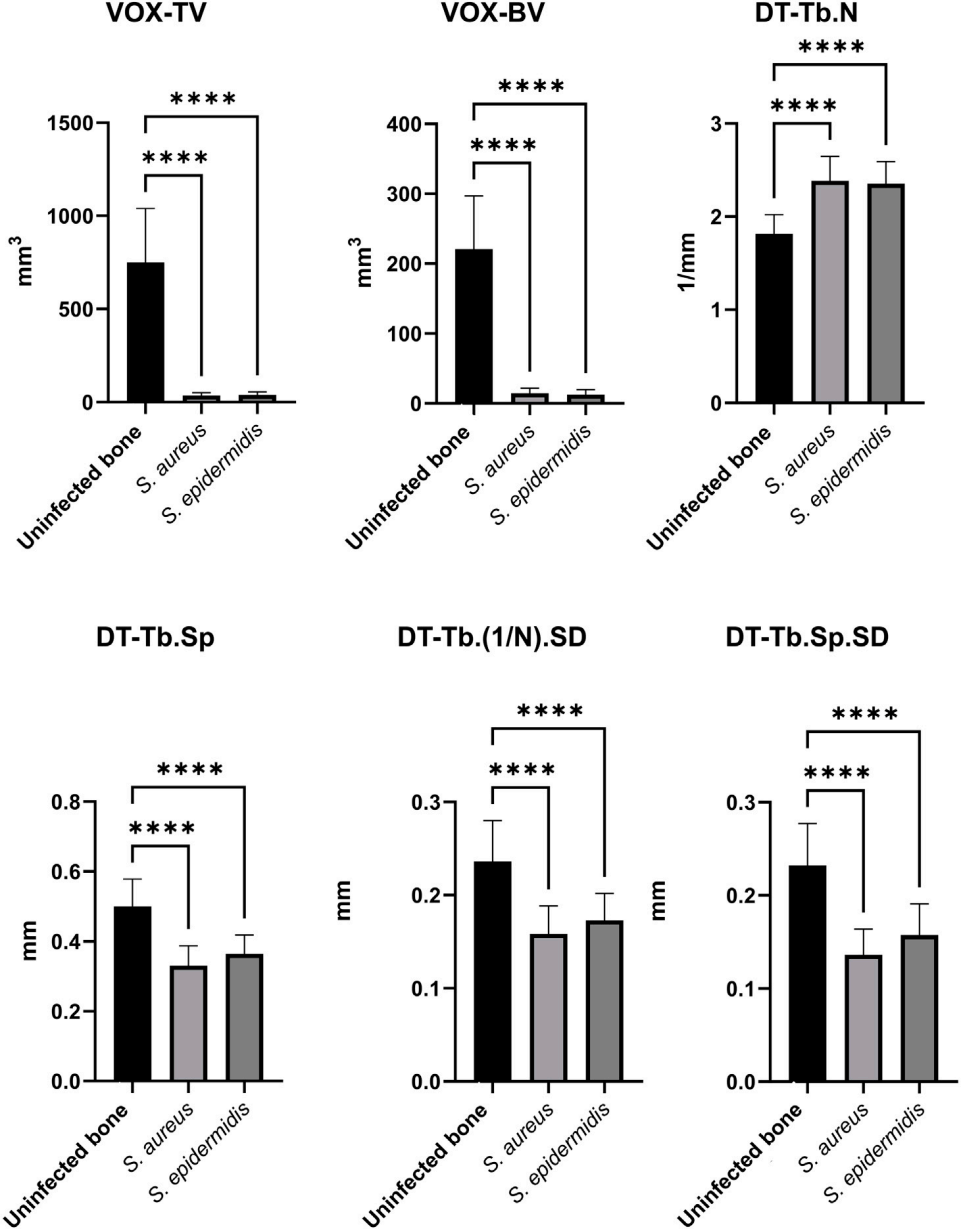
Comparisons of key micro-CT structural markers differentiating human non-infected from infected bone samples. The bar plots present mean values ± standard deviations of uninfected bone (as control), *S. aureus*-inoculated bone, and *S. epidermidis*-inoculated bone. Significance was assessed using one-way ANOVA, with * (p < 0.05), ** (p < 0.01), *** (p < 0.001), and **** (p < 0.0001).

Micro-CT enables structural assessment of bone tissues, with Figure 7 presenting representative cross-sectional micro-CT scans for the three experimental groups: uninfected bone, *S. aureus*-inoculated bone, and *S. epidermidis*-inoculated. As illustrated in Figure 8, each group demonstrated distinct results in 3D segmentation, trabecular thickness, and trabecular separation. One-way ANOVA was performed to evaluate the diagnostic potential of micro-CT-derived structural markers for distinguishing between inoculated and uninfected bone tissue (Figure 9). Several parameters, including total volume (VOX-TV), bone volume (VOX-BV), trabecular number (DT-Tb.N), trabecular separation (DT-Tb.Sp), its standard deviation (DT-Tb.Sp.SD), and intra-individual variation of trabecular number (DT-Tb.(1/N).SD), showed highly significant differences (p < 0.0001).

Certain parameters appear helpful in distinguishing between uninfected and *S. aureus*-inoculated bone, but are less effective for differentiating *S. epidermidis*-inoculated bone from uninfected bone. Parameters such as trabecular connectivity (Conn-Dens), trabecular shape (TRI-SMI), mean trabecular thickness (DT-Tb.Th), intra-individual variation of trabecular thickness (DT-Tb.Th.SD), and the mean value of the segmented region (Mean2) showed no or only low statistical significance for both pathogens and are therefore considered less suitable for distinguishing between inoculated and uninfected bone samples (data not presented). Interestingly, the higher significance of specific parameters in *S. epidermidis*-inoculated bone compared to *S. aureus*-inoculated bone suggests that the two pathogens differ in how they impact bone structure.

### 3.5 Comparison of micro-CT parameters between bone samples infected with *S. aureus* and *S. epidermidis*

**FIGURE 10:**
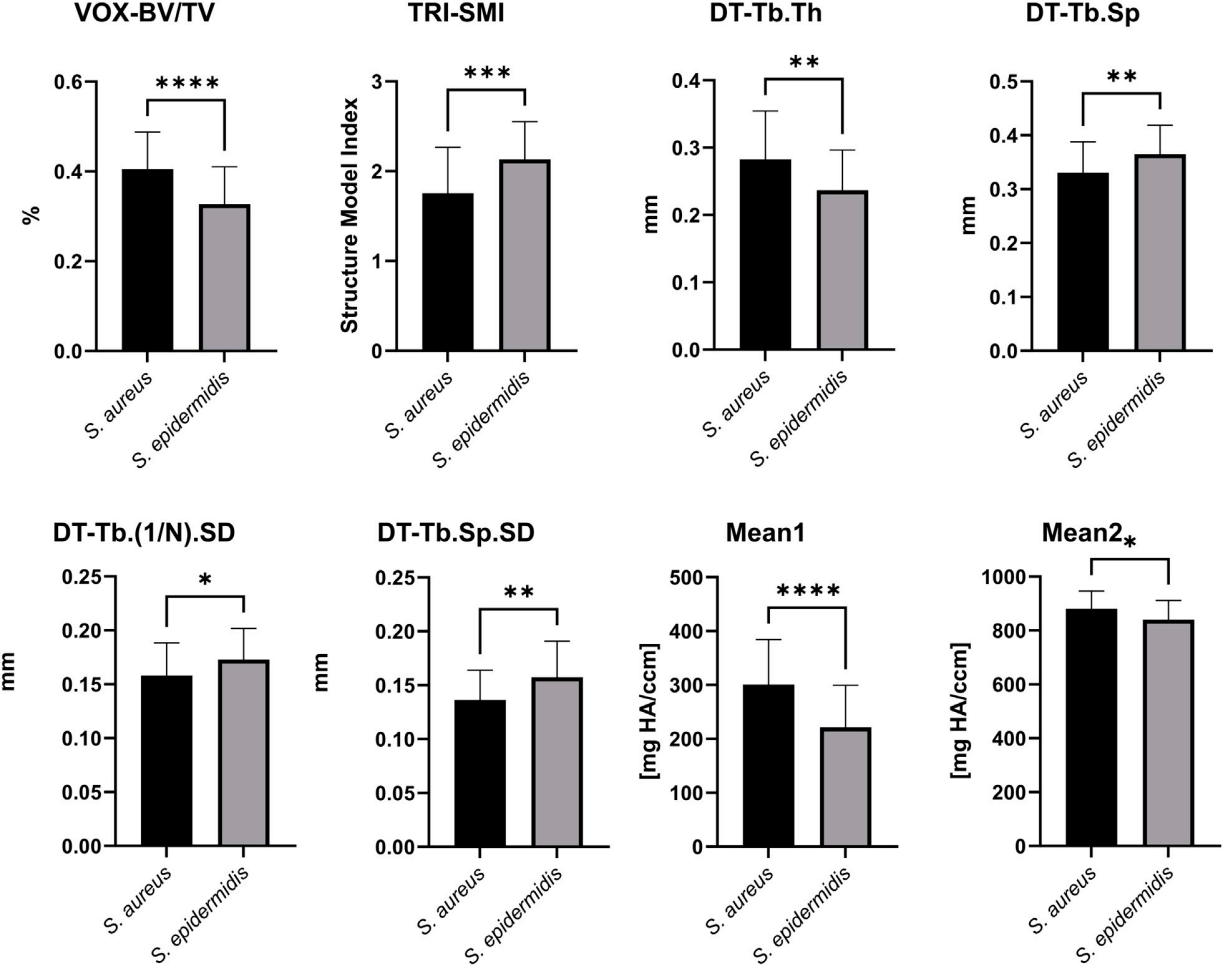
Comparison of micro-CT parameters between *S. aureus*- and *S. epidermidis*-inoculated bone samples. The bar plots present mean values ±standard deviation for key micro-CT structural markers, highlighting pathogen-specific differences. Analyzed parameters include VOX-BV/TV, TRI-SMI, DT-Tb.Th, DT-Tb.Sp, DT-Tb.(1/N).SD, DT-Tb.Sp.SD, Mean1 and Mean2. Statistical significance was determined using a two-tailed t-test, with p-values indicated as follows: * (p < 0.05), ** (p < 0.01), *** (p < 0.001), and **** (p < 0.0001).

The next objective was to achieve pathogen-specific differentiation using micro-CT-derived markers. The results of the two-tailed t-test are presented in Figure 10. Significant differences between bone samples inoculated with *S. aureus* and *S. epidermidis* were observed for trabecular thickness (DT-Tb.Th), mean trabecular separation (DT-Tb.Sp), and its standard deviation (DT-Tb.Sp.SD). Additionally, the structure model index (TRI-SMI) contributed to differentiating between the two groups. The most informative markers were the ratio of trabecular volume to total volume (VOX-BV/TV) and the mean voxel values (Mean1), both of which showed high statistical significance (p < 0.0001). In contrast, parameters such as total volume (VOX-TV) and trabecular volume (VOX-BV) did not distinguish the two bacterial strains clearly.

### 3.6 Comparison of Raman microscopy, micro-CT, and their combination for SVM-Based classification of bone inoculation status

To evaluate the diagnostic performance of Raman spectroscopy markers, micro-CT parameters, and their combination, Support Vector Machine (SVM) classifiers were trained and evaluated using repeated stratified k-fold cross-validation (5 folds, 10 repeats). Two binary classification tasks were analyzed: (1) inoculated vs. uninfected bone samples, and (2) bone samples inoculated with *S. aureus* vs. *S. epidermidis*.

**FIGURE 11:**
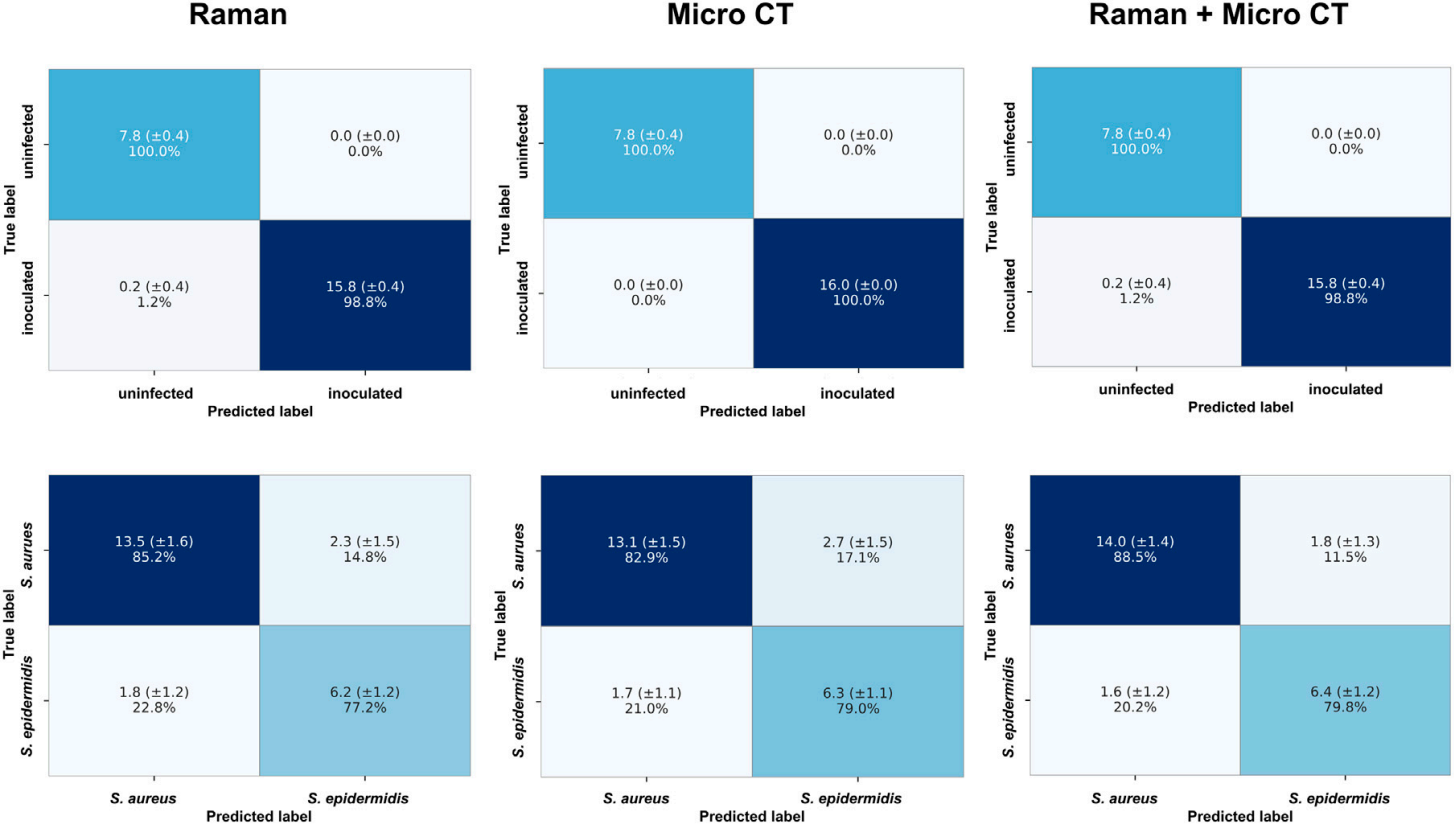
Aggregated confusion matrices summarizing SVM classification performance using repeated stratified k-fold cross-validation (5 folds, 10 repeats). Models were trained using Raman spectroscopy markers (left column), micro-CT parameters (middle column), or a combination of both (right column). The top row shows results for the binary classification of inoculated vs. uninfected bone samples; the bottom row displays results for classifying samples inoculated with *S. aureus* vs. *S. epidermidis*. Values represent the mean number of samples correctly or incorrectly classified, with standard deviation (±SD) across cross-validation runs, and row-wise percentages.

Aggregated confusion matrices summarizing classification outcomes are presented in Figure 11, and detailed performance metrics are reported in Table 3.

**TABLE 3:** Comparison of classification performance metrics (mean values across 50 runs) for SVM models trained to distinguish (left inoculated vs. uninfected bone samples and (right) bone samples inoculated with *S. aureus* and *S. epidermidis* infections (right). Models were trained using either Raman spectroscopy markers features, micro-CT parameters only, or a combination of both. Performance metrics include mean accuracy, precision, recall, and F1-score, calculated from repeated stratified k-fold cross-validation (5 folds, 10 repeats).

Model	Raman only	Micro-CT only	Raman + microCT	Raman only	microCT only	Raman + microCT
Classification task	Inoculated vs. Uninfected			*S. aureus* vs. *S. epidermidis*		
Mean Accuracy [%]	99.16	100.00	99.16	82.51	81.61	85.55
Mean Precision [%]	1.00	1.00	1.00	73.95	72.00	79.57
Mean Recall [%]	98.75	1.00	98.75	77.25	79.00	79.75
Mean F1 [%]	99.35	1.00	99.35	74.83	74.29	78.54

In the incouclated vs. uninfected task, both Raman-derived biochemical markers and micro-CT-based structural parameters yielded near-perfect classification results. The Raman-only model achieved an average accuracy of 99.16%. The micro-CT-only model achieved 100% accuracy, with perfect performance across metrics. The combined model also reached 99.16% accuracy, suggesting that Raman spectroscopy alone provided sufficient discriminatory power in this task. The addition of micro-CT features did not improve performance, likely due to the ceiling effect in classification accuracy.

In the more challenging task of distinguishing *S. aureus* from S. epidermidis, the combined Raman + micro-CT model achieved higher accuracy than either unimodal models. The Raman- only and micro-CT-only classifiers reached average accuracies of 82.51% and 81.61%, respectively. The combined model achieved 85.55% accuracy, with corresponding improvements in precision, recall, and F1-score. A Wilcoxon signed-rank test confirmed that this accuracy was statistically significant (p < 0.05) compared to both single-modality models (p = 0.0179 vs. Raman-only; p = 0.0035 vs. micro-CT-only). The combination of Raman and micro-CT data resulted in improved classification performance compared to single-modality models, with statistically significant gains in accuracy.

### 3.7 Principal component analysis (PCA) and feature contributions of combined Raman spectroscopy markers and micro-CT parameters

**FIGURE 12:**
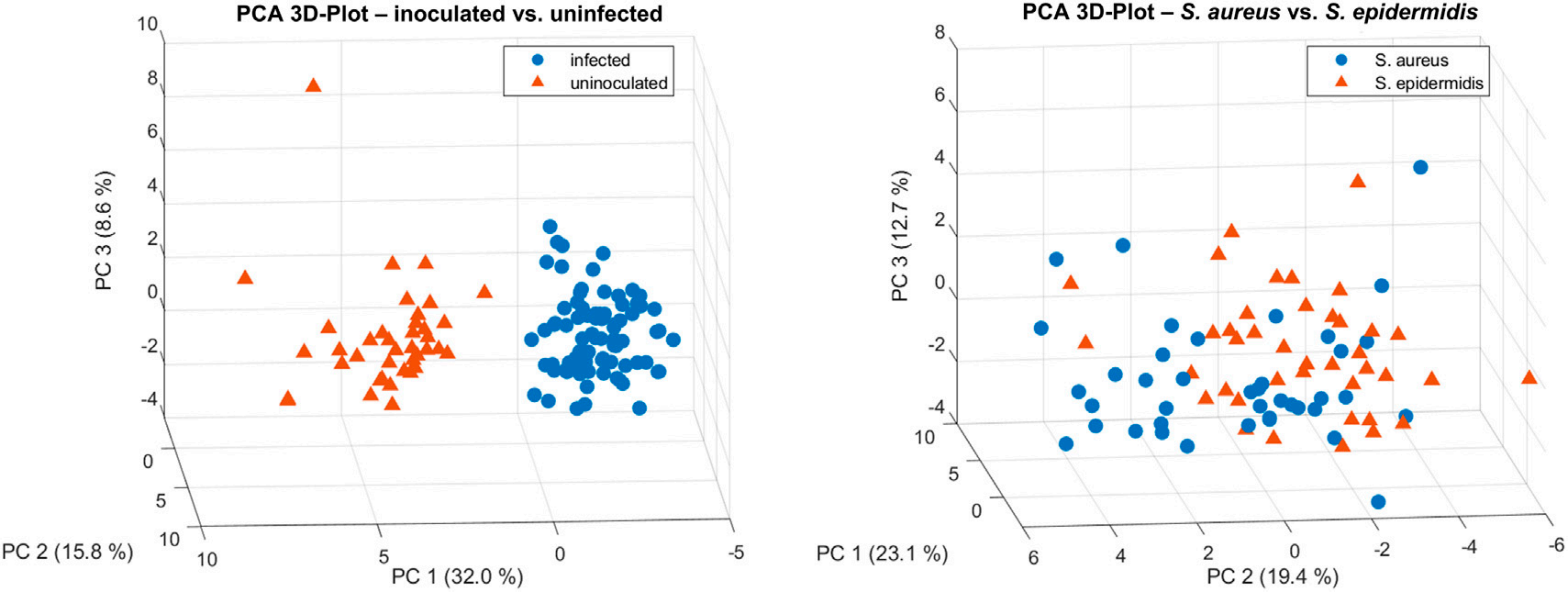
3D PCA plots based on combined Raman spectroscopy markers and micro-CT parameters for characterization of bone samples. Left: PCA distinguished between inoculated and uninfected bone samples, indicating clear separation along principal components. Right: PCA reveals overlapping clusters for samples inoculated with either *S. aureus* or *S. epidermidis*, suggesting only limited species-specific differentiation.

**FIGURE 13:**
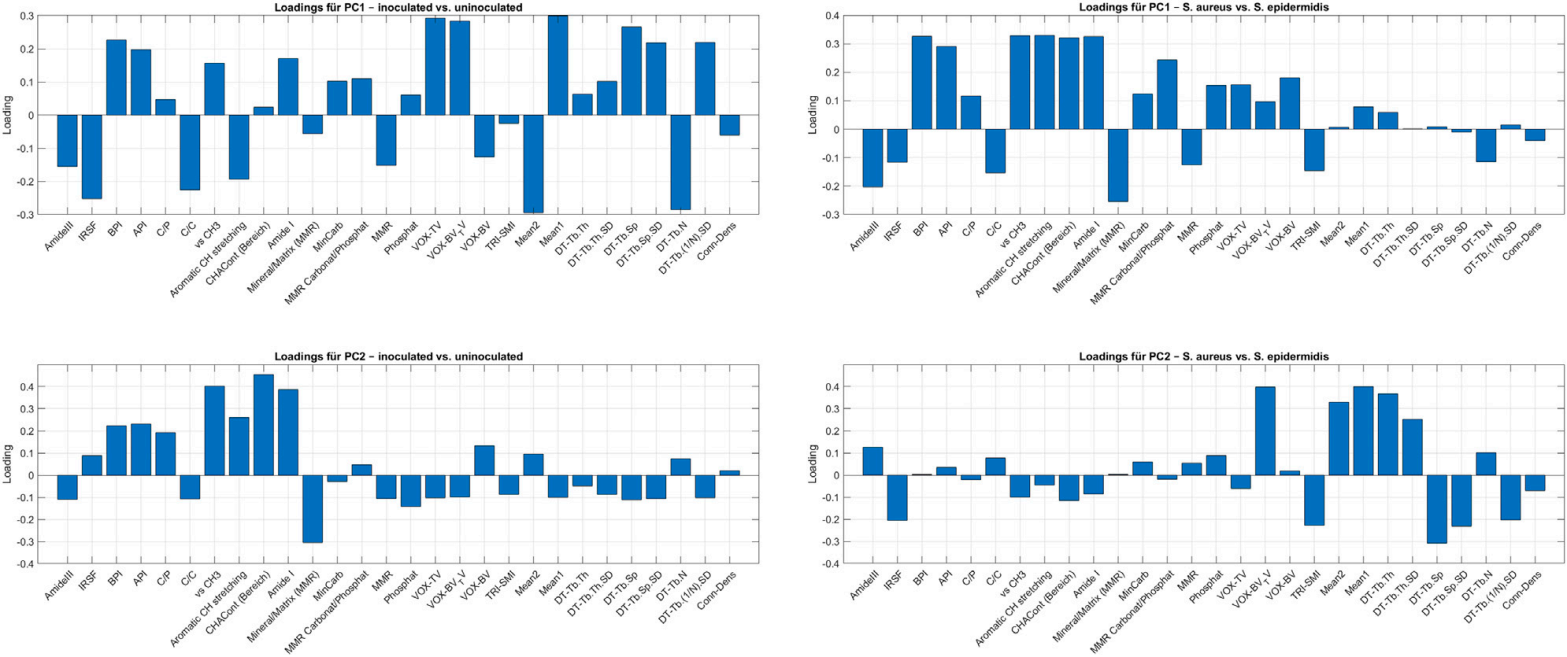
Loadings plots from PCA performed on combined Raman spectroscopy markers and micro-CT parameters to explore relevant features in the differentiation of bone samples. The top two plots show loadings for PC1 and PC2 from the PCA comparing inoculated and uninfected samples. The bottom two plots show loadings for PC1 and PC2 from the PCA comparing bone samples inoculated with either *S. aureus* or *S. epidermidis*. Loadings indicate the contribution of each variable to the respective principal component and serve to identify features most associated with variance in inoculation status.

To identify the most relevant features contributing to the variance in the dataset, PCA was performed using the combined Raman markers and micro-CT parameters. The 3D PCA plots in Figure 12 illustrate the overall structure of the data. A clear separation between inoculated and uninfected bone samples was observed along the first two principal components (PC1 and PC2), whereas the distributions of samples inoculated with either *S. aureus* or *S. epidermidis* showed considerable overlap (Figure 12). To further interpret the sources of variance, PCA loadings plots were analyzed (Figure 13).

For the inoculated vs. uninfected comparison, PC1 was dominated by a mix of Raman and micro-CT features, including Mean1, Mean2, VOX-TV, DT-Tb.N, VOX-BV/TV, IRSF, and BPI. In contrast, PC2 highlighted Raman-derived biochemical markers such as CHACont, vs CH_3_, Amide I, MMR, and Aromatic CH stretching.

For the *S. aureus* vs. *S. epidermidis* comparison, PC1 was strongly influenced by Raman spectral features (Aromatic Ch stretching, vs CH_3_, Amide I, MMR Carbonat/Phosphat, API, BPI), while PC2 was primarily shaped by structural parameters derived from micro-CT (Mean1, DT-Tb-Th, DT-Tb.Sp, TRI-SMI, IRSF, DT-Tb. (1/N).SD).

## 4 Discussion

This study demonstrates the added diagnostic value of integrating Raman microscopy and micro-CT imaging for detecting and characterizing bone infections. By combining these techniques, we obtained comprehensive insights into the molecular composition, mineralization patterns, and structural integrity of inoculated bones, allowing precise evaluation of infection-induced bone degradation.

The findings confirm that bacterial infections cause a substantial loss of mineral (phosphate and carbonate) and organic (collagen, lipid) components in bone at both the molecular and microstructural levels.

Raman spectroscopy effectively distinguished infected from uninfected bone, showing significant reductions in phosphate (v_1_PO_4_^3−^), carbonate, Amide I, and Amide III bands in infected samples. Key Raman markers for bone degradation included IRSF, BPI, API, C/P, C/C, phosphate, crystallinity, and MMR. PCA identified v_1_PO_4_^3−^, Amide III, and CH_2_ deformation as the most reliable molecular indicators of infection-related changes. Pathogen-specific differentiation between *S. aureus* and *S. epidermidis* was less distinct, although differences in v_1_PO_4_^3−^ phosphate, Amide III, aromatic CH stretching, and mineral carbonate content suggested some species-specific molecular effects. Previous studies suggest that these molecular differences may be related to the distinct biofilm-forming capabilities and enzyme production profiles of these species, affecting osteogenic differentiation and bone remodeling differently (Tübel et al., 2021).

Micro-CT imaging revealed apparent structural differences between inoculated and uninfected bone, particularly in bone volume, trabecular number, and separation. The most significant micro-CT markers included VOX-TV, VOX-BV, DT-Tb.N, DT-Tb.Sp, and DT-Tb.Sp.SD. Differentiation between *S. aureus* and *S. epidermidis* was most successful using VOX-BV/TV and Mean1, both exhibiting high significance (p < 0.0001). These structural variations might reflect different remodeling patterns driven by species-specific biofilm formation, virulence factors, and interactions with bone matric components. Specifically, *S. aureus* is known for its aggressive invasiveness and toxin production, causing rapid osteolysis, whereas *S. epidermidis* generally exhibits more subtle effects, characterized by a reduction in mineralization and altered osteoblast activity (Tübel et al., 2021; Wright and Nair, 2010).

By integrating molecular and structural data, correlations between Raman and micro-CT markers were established. Phosphate and carbonate Raman markers correlated with bone volume and trabecular number, suggesting a link between mineral composition and bone density. Similarly, Amide I and Amide III bands were associated with trabecular separation and relative bone volume, indicating that collagen degradation contributes to structural weakening. These findings underscore the complementary nature of Raman microscopy and micro-CT imaging, offering a more comprehensive evaluation of bone quality and infection-related changes. Our findings align with recent studies employing multiple spectroscopic methods, including Brillouin–Raman microspectroscopy and ATR-FTIR spectroscopy, which showed substantial biochemical and mechanical changes in bone following *S. aureus* infection. Similar to our observations, these studies highlighted significant reductions in lipid and protein content alongside alterations in mineralization patterns, indicating extensive pathogen-induced bone remodeling and matrix degradation (Alunni Cardinali et al., 2024).

The potential added value of combining Raman and micro-CT was explored through classification performance usingSVMs evaluated with repeated stratified k-fold cross-validation. In the task of distinguishing between inoculated vs. uninfected bone samples, both Raman-derived biochemical markers and micro-CT structural parameters individually achieved near-perfect classification performance (mean accuracies of 99.16% and 100%, respectively). These results indicates that either modality alone can robustly detect infection in this binary context, and combining them did not further improve overall accuracy.

For the more challenging task of differentiating bone sample inoculated with *S. aureus* from bone samples inoculated with *S. epidermidis*, classification performance was lower overall, but a modest improvement was observed when combining both data types. The Raman-only and micro-CT-only models yielded mean accuracies of 82.51% and 81.61%, respectively, whereas the combined model reached 85.55%. Although the absolute increase was limited, the improvement was statistically significant based on Wilcoxon signed-rank tasts (p < 0.05) comparing accuracy distributions of the combined model to each unimodal baseline across 50 cross-validation runs.

These findings suggest, that while individual modalities already capture major infection-related alterations in bone, combing structural and molecular information may provide complementary input that supports more consistent classification in subtler pathogen-specific scenarios. The observed performance gain, though moderate, highlights the potential diagnostic benefit of multimodal pproaches in tasks where discrimatice features are distributed across different biological scales.

Certain Raman markers, such as the CH stretching bands and Amide III, enabled differentiation between *S. aureus* and *S. epidermidis*, indicating their potential as pathogen-specific diagnostic markers (Figure 1). Raman microscopy proved highly sensitive for distinguishing inoculated from uninfected bone, consistent with previous studies on mineralized tissues (Goswami et al., 2018; Beck-Broichsitter et al., 2015). In this study, reductions in phosphate (v_1_PO_4_^3−^), carbonate, Amide I, and Amide III bands suggest a pathogen-induced degradation of the mineral and organic matrix (Pallua et al., 2012; Pallua et al., 2017; Mozhaeva et al., 2022). Additionally, Mohamed Khalid et al. demonstrated using Raman spectroscopy that *S. aureus* infection significantly reduced bone mineral quality and crystallinity and altered collagen cross-linking, indicating the capability of Raman spectroscopy for early and rapid diagnosis of staphylococcal osteomyelitis. They observed substantial reductions in phosphate and carbonate mineral-to-matrix ratios (MMR) and significant changes in collagen structure, which were consistent with our findings, further supporting the sensitivity and diagnostic potential of Raman spectroscopy for bone infections (Khalid et al., 2018). Moreover, the persistence of intracellular *S. aureus* in osteoblasts, osteoclasts, and osteocytes, as reviewed by Kareme D. Alder et al., provides a potential explanation for recurring infections and continuous inflammatory damage in osteomyelitis. This intracellular presence can induce prolonged inflammatory responses, enhancing osteoclastogenesis and further aggravating bone loss. This phenomenon underscores the importance of multimodal diagnostic approaches capable of detecting not only extracellular pathogens but also subtle intracellular pathogen effects that contribute to chronicity and recurrence in bone infections (Alder et al., 2020). These observations are Key Raman parameters – including IRSF, BPI, API, C/P, C/C, phosphate, crystallinity, and the MMR – emerged as reliable markers for differentiating inoculated from uninfected bone. These results align with previous reports emphasizing phosphate crystallinity and carbonate substitutions as essential indicators of bone pathology (Backhaus et al., 2016; Kessler, 2011). However, while infection-induced changes were detectable, pathogen-specific differentiation between *S. aureus* and *S. epidermidis* was less pronounced. Significant spectral differences were mainly observed in v_1_PO_4_^3−^ phosphate, Amide III, aromatic CH stretching, and mineral carbonate content, suggesting subtle variations in how these bacteria affect bone metabolism (Kessler, 2011). These findings support previous observations that Raman-based identification of specific bacteria in bone remains challenging due to overlapping spectral changes associated with general infection-related inflammation and degradation processes (Goswami et al., 2018; Beck-Broichsitter et al., 2015). These observations are further supported by the PCA loading plots (Figure 13), which identify the key Raman and micro-CT features contributing to variance in the dataset. For infection status, PC1 was dominated by both spectroscopic (IRSF, BPI, Mean1) and micro-CT (VOX-TV, DT-Tb.N, VOX-BV/TV) parameters. For species differentiation, PC1 emphasized Raman features (Amide I, Aromatic CH stretching, CH_2_ deformation), while PC2 highlighted structural features (DT-Tb.Sp, DT-Tb.Th, TRI-SMI). This multimodal feature distribution reinforces the idea that biochemical changes dominate infection detection, while subtle structural shifts contribute to species-specific differentiation.

The micro-CT analysis revealed significant structural differences between uninfected and inoculated bone, particularly in trabecular bone parameters (Goswami et al., 2018). Notably, differentiation between *S. aureus* and *S. epidermidis* was most successful using VOX-BV/TV and Mean1 (both p < 0.0001). This suggests that different bacterial species may induce distinct remodeling patterns, with *S. epidermidis* potentially causing more pronounced trabecular thinning and loss of connectivity (Backhaus et al., 2016; Kessler, 2011). These structural changes align with previous reports of infection-driven bone resorption and porosity increases (Goswami et al., 2018; Beck-Broichsitter et al., 2015). A significant advantage of this study was the ability to correlate molecular and structural markers, bridging the gap between biochemical composition and physical bone integrity. The observed correlation between phosphate and carbonate Raman markers with bone volume and trabecular number supports the hypothesis that bone mineral loss is directly linked to trabecular thinning (Pallua et al., 2012; Pallua et al., 2017; Mozhaeva et al., 2022). Similarly, the correlation between Amide I and Amide III bands with trabecular separation and relative bone volume suggests that collagen degradation contributes to trabecular instability (Backhaus et al., 2016). These findings reinforce previous studies highlighting the importance of collagen integrity in maintaining bone strength and suggest that a combined Raman-micro-CT approach could enhance infection diagnostics by providing a dual perspective on bone health (Goswami et al., 2018; Beck-Broichsitter et al., 2015). While Raman microscopy identifies early-stage molecular changes, micro-CT captures advanced structural deterioration, complementing these two modalities. Integrating both methods could significantly improve infection diagnostics and risk assessment for bone graft procedures (Goswami et al., 2018). Identifying distinct Raman and micro-CT markers may also support real-time evaluation of infection status, potentially informing surgical decision-making in orthopaedic and trauma surgery (Pallua et al., 2012; Pallua et al., 2017; Mozhaeva et al., 2022).

The *ex vivo* setting employed in this study provided an ideal environment for controlled and precise analysis of infection-induced changes at the molecular and structural levels. However, this model does not fully replicate the complex immunological responses, vascular dynamics, and systemic interactions present in clinical bone infections. Consequently, the extent to which these findings can be directly translated into clinical practice warrants further investigation. Additionally, clinical scenarios frequently involve factors such as prior antibiotic treatment, varying pathogen load, host immune status, and chronicity of infection, all of which significantly influence disease progression and bone remodeling patterns. Future research should incorporate these clinical variables to evaluate the robustness and generalizability of Raman spectroscopy and micro-CT imaging as diagnostic tools for bone infections under more realistic clinical conditions. Future research should focus on expanding pathogen-specific spectral libraries, refining automated classification models, and integrating machine-learning approaches for more accurate, high-throughput diagnostics (Kessler, 2011). Incorporating larger samples sizes and longitudinal designs will also be essential to validate diagnostic potential across different infection stages. Overall, the results of this study highlight the potential of combining Raman microscopy and micro-CT imaging for a more precise, multi-scale assessment of bone infection - ultimately contributing to improved diagnostic accuracy and patient outcomes in clinical settings.

## 5 Conclusion

This study highlights the power of Raman microscopy and micro-CT imaging for the detailed characterization of bone tissue. Raman microscopy provided molecular insights into bone composition, detecting significant reductions in phosphate, carbonate, and collagen-related bands in inoculated samples. Meanwhile, micro-CT imaging captured structural alterations, particularly in trabecular number, separation, and bone volume, enabling a quantitative assessment of infection-induced bone degradation. While both techniques effectively differentiated inoculated from uninfected bone, the pathogen-specific discrimination between *S*. *aureus* and *S*. *epidermidis* was less distinct in Raman analysis but more evident in micro-CT parameters such as VOX-BV/TV and Mean1. These findings suggest that different bacterial species induce varying patterns of bone degradation, which could be further explored in future research. Importantly, supervised classification using SVM models confirmed that combining Raman and micro-CT features yielded additive diagnostic value—particularly in distinguishing between bacterial species, where the combined model moderately improved classification performance compared to single-modality approaches. The integration of early-stage molecular markers and later-stage structural changes supports a dual-scale diagnostic framework. PCA further revealed that Raman and micro-CT features contributed distinctly to variance in infection and pathogen-specific groupings. Loadings from PC1 and PC2 demonstrated that phosphate, Amide III, and mineral ratios were dominant biochemical markers, while trabecular metrics were key structural contributors. These analyses help elucidate which features are most biologically informative and may serve as targets for future diagnostic model refinement. In addition, correlations between molecular and structural parameters—such as phosphate and carbonate content with bone volume and trabecular number, and Amide I/III bands with trabecular separation—demonstrated that biochemical degradation is tightly linked to loss of mechanical bone integrity. This underscores the value of multimodal integration for a more holistic understanding of bone infection pathophysiology. Integrating molecular and structural data offers a more comprehensive approach to assessing bone quality, infection status, and potential suitability for transplantation. By combining early-stage biochemical alterations detected by Raman microscopy with micro-CT-based structural assessment, this dual-modality approach could improve diagnostic precision in clinical and research applications. While species-specific differentiation remains challenging using Raman spectroscopy alone, the combined approach provides enhanced resolution and may be critical for detecting subtle pathogen-dependent remodeling effects. Continued efforts to optimize feature integration and account for temporal infection dynamics will further strengthen its diagnostic relevance. Future studies should focus on expanding pathogen-specific spectral databases, refining automated classification models, and incorporating machine learning algorithms to enhance real-time, high-throughput diagnostics. Ultimately, this integrative strategy may contribute to more accurate infection risk assessment, optimized bone graft selection, and improved orthopaedic and trauma surgery outcomes.

## Data Availability

The raw data supporting the conclusions of this article will be made available by the authors, without undue reservation.

## References

[B1] Alder K. D. Lee I. Munger A. M. Kwon H. K. Morris M. T. Cahill S. V. (2020). Intracellular *Staphylococcus aureus* in bone and joint infections: a mechanism of disease recurrence, inflammation, and bone and cartilage destruction. Bone 141, 115568. 10.1016/j.bone.2020.115568 32745687

[B2] Alunni Cardinali M. Govoni M. Stefani S. Maso A. Storni E. Valenti F. (2024). Combining multiple spectroscopic techniques to reveal the effects of *Staphylococcus aureus* infection on human bone tissues. Appl. Spectrosc. 78 (12), 1295–1306. 10.1177/00037028241278903 39344289

[B3] Backhaus K. Backhaus K. Erichson B. Plinke W. Weiber R. (2016). Multivariate analysemethoden. Springer.

[B4] Beck-Broichsitter B. E. Smeets R. Heiland M. (2015). Current concepts in pathogenesis of acute and chronic osteomyelitis. Curr. Opin. Infect. Dis. 28 (3), 240–245. 10.1097/qco.0000000000000155 25918958

[B5] Bezstarosti H. Van Lieshout E. M. M. Voskamp L. W. Kortram K. Obremskey W. McNally M. A. (2019). Insights into treatment and outcome of fracture-related infection: a systematic literature review. Arch. Orthop. Trauma Surg. 139 (1), 61–72. 10.1007/s00402-018-3048-0 30343322 PMC6342870

[B6] Boskey A. L. Imbert L. (2017). Bone quality changes associated with aging and disease: a review. Ann. N. Y. Acad. Sci. 1410 (1), 93–106. 10.1111/nyas.13572 29265417 PMC5774017

[B7] Bury D. C. Rogers T. S. Dickman M. M. (2021). Osteomyelitis: diagnosis and treatment. Am. Fam. Physician 104 (4), 395–402. 10.7759/cureus.r144 34652112

[B8] Chen W. L. Chang W. N. Chen Y. S. Hsieh K. S. Chen C. K. H. Peng N. J. (2010). Acute community-acquired osteoarticular infections in children: high incidence of concomitant bone and joint involvement. J. Microbiol. Immunol. Infect. 43 (4), 332–338. 10.1016/s1684-1182(10)60051-5 20688294

[B9] Cooper D. Turinsky A. Sensen C. Hallgrimsson B. (2007). Effect of voxel size on 3D Micro-CT analysis of cortical bone porosity. Calcif. Tissue Int. 80 (3), 211–219. 10.1007/s00223-005-0274-6 17340226

[B10] DePaula C. A. Truncale K. Gertzman A. Sunwoo M. Dunn M. (2005). Effects of hydrogen peroxide cleaning procedures on bone graft osteoinductivity and mechanical properties. Cell Tissue Bank. 6 (4), 287–298. 10.1007/s10561-005-3148-2 16308768

[B11] Dym H. Zeidan J. (2017). Microbiology of acute and chronic osteomyelitis and antibiotic treatment. Dent. Clin. North Am. 61 (2), 271–282. 10.1016/j.cden.2016.12.001 28317566

[B12] Eastlund T. (2006). Bacterial infection transmitted by human tissue allograft transplantation. Cell Tissue Bank. 7 (3), 147–166. 10.1007/s10561-006-0003-z 16933037

[B13] France C. A. M. Sugiyama N. Aguayo E. (2020). Establishing a preservation index for bone, dentin, and enamel bioapatite mineral using ATR-FTIR. J. Archaeol. Sci. Rep. 33, 102551. 10.1016/j.jasrep.2020.102551

[B14] Freeman J. J. Wopenka B. Silva M. Pasteris J. (2001). Raman spectroscopic detection of changes in bioapatite in mouse femora as a function of age and *in vitro* fluoride treatment. Calcif. Tissue Int. 68 (3), 156–162. 10.1007/s002230001206 11351499

[B15] Goswami K. Parvizi J. Maxwell Courtney P. (2018). Current recommendations for the diagnosis of acute and chronic PJI for hip and knee-cell counts, alpha-Defensin, leukocyte esterase, next-generation sequencing. Curr. Rev. Musculoskelet. Med. 11 (3), 428–438. 10.1007/s12178-018-9513-0 30062484 PMC6105482

[B16] Grieb T. A. Forng R. Y. Stafford R. E. Lin J. Almeida J. Bogdansky S. (2005). Effective use of optimized, high-dose (50 kGy) gamma irradiation for pathogen inactivation of human bone allografts. Biomaterials 26 (14), 2033–2042. 10.1016/j.biomaterials.2004.06.028 15576177

[B17] Grunenwald A. Keyser C. Sautereau A. Crubézy E. Ludes B. Drouet C. (2014). Revisiting carbonate quantification in apatite (bio) minerals: a validated FTIR methodology. J. Archaeol. Sci. 49, 134–141. 10.1016/j.jas.2014.05.004

[B18] Hartman B. J. Tomasz A. (1984). Low-affinity penicillin-binding protein associated with beta-lactam resistance in Staphylococcus aureus. J. Bacteriol. 158 (2), 513–516. 10.1128/jb.158.2.513-516.1984 6563036 PMC215458

[B19] Hinsenkamp M. Muylle L. Eastlund T. Fehily D. Noël L. Strong D. M. (2012). Adverse reactions and events related to musculoskeletal allografts: reviewed by the World Health Organisation Project NOTIFY. Int. Orthop. 36 (3), 633–641. 10.1007/s00264-011-1391-7 22048753 PMC3291755

[B20] Izakovicova P. Borens O. Trampuz A. (2019). Periprosthetic joint infection: current concepts and outlook. EFORT Open Rev. 4 (7), 482–494. 10.1302/2058-5241.4.180092 31423332 PMC6667982

[B21] Kessler W. (2011). Multivariate datenanalyse: für die pharma, bio-und Prozessanalytik. John Wiley and Sons.

[B22] Khalid M. Bora T. Ghaithi A. A. Thukral S. Dutta J. (2018). Raman spectroscopy detects changes in bone mineral quality and collagen cross-linkage in staphylococcus infected human bone. Sci. Rep. 8 (1), 9417. 10.1038/s41598-018-27752-z 29925892 PMC6010429

[B23] Lentino J. R. (2003). Prosthetic joint infections: bane of orthopedists, challenge for infectious disease specialists. Clin. Infect. Dis. 36 (9), 1157–1161. 10.1086/374554 12715311

[B24] Lew D. P. Waldvogel F. A. (2004). *Osteomyelitis.* Lancet 364 (9431), 369–379. 10.1016/s0140-6736(04)16727-5 15276398

[B25] Lewis C. S. Katz J. Baker M. I. Supronowicz P. R. Gill E. Cobb R. R. (2011). Local antibiotic delivery with bovine cancellous chips. J. Biomater. Appl. 26 (4), 491–506. 10.1177/0885328210375729 20819915

[B26] Lüllmann-Rauch R. Paulsen F. (2012). Taschenlehrbuch Histologie. Thieme.

[B27] McCreadie B. R. Morris M. D. Chen T. c. Sudhaker Rao D. Finney W. F. Widjaja E. (2006). Bone tissue compositional differences in women with and without osteoporotic fracture. Bone 39 (6), 1190–1195. 10.1016/j.bone.2006.06.008 16901772

[B28] Mandair G. S. Morris M. D. (2015). Contributions of Raman spectroscopy to the understanding of bone strength. Bonekey Rep. 4, 620. 10.1038/bonekey.2014.115 25628882 PMC4296861

[B29] Metsemakers W. J. Kuehl R. Moriarty T. Richards R. Verhofstad M. Borens O. (2018). Infection after fracture fixation: current surgical and microbiological concepts. Injury 49 (3), 511–522. 10.1016/j.injury.2016.09.019 27639601

[B30] Morris M. D. Mandair G. S. (2011). Raman assessment of bone quality. Clin. Orthop. Relat. Res. 469 (8), 2160–2169. 10.1007/s11999-010-1692-y 21116756 PMC3126952

[B31] Mozhaeva V. Kudryavtsev D. Prokhorov K. Utkin Y. Gudkov S. Garnov S. (2022). Toxins' classification through Raman spectroscopy with principal component analysis. Spectrochim. Acta A Mol. Biomol. Spectrosc. 278, 121276. 10.1016/j.saa.2022.121276 35504103

[B32] Ostertag A. Peyrin F. Fernandez S. Laredo J. D. de Vernejoul M. C. Chappard C. (2014). Cortical measurements of the tibia from high resolution peripheral quantitative computed tomography images: a comparison with synchrotron radiation micro-computed tomography. Bone 63, 7–14. 10.1016/j.bone.2014.02.009 24582804

[B33] Pallua J. D. Pezzei C. Zelger B. Schaefer G. Bittner L. K. Huck-Pezzei V. A. (2012). Fourier transform infrared imaging analysis in discrimination studies of squamous cell carcinoma. Analyst 137 (17), 3965–3974. 10.1039/c2an35483g 22792538

[B34] Pallua J. D. Unterberger S. H. Pemberger N. Woess C. Ensinger C. Zelger B. (2017). Retrospective case study on the suitability of mid-infrared microscopic imaging for the diagnosis of mucormycosis in human tissue sections. Anal. Methods 9 (28), 4135–4142. 10.1039/c7ay01132f

[B35] Patel R. (2023). Periprosthetic joint infection. N. Engl. J. Med. 388 (3), 251–262. 10.1056/nejmra2203477 36652356

[B36] Perka C. Haas N. (2011). Periprosthetic infection. Der Chir. 82, 218–226. 10.1007/s00104-010-2014-3 21340589

[B37] Pineda C. Vargas A. Rodríguez A. V. (2006). Imaging of osteomyelitis: current concepts. Infect. Dis. Clin. North Am. 20 (4), 789–825. 10.1016/j.idc.2006.09.009 17118291

[B38] Recker R. R. Marin F. Ish-Shalom S. Möricke R. Hawkins F. Kapetanos G. (2009). Comparative effects of teriparatide and strontium ranelate on bone biopsies and biochemical markers of bone turnover in postmenopausal women with osteoporosis. J. Bone Mineral Res. 24 (8), 1358–1368. 10.1359/jbmr.090315 19338452

[B39] Rodrigues C. R. F. Oliveira J. Fulco U. Albuquerque E. Moura R. Caetano E. (2013). Quantum biochemistry study of the T3-785 tropocollagen triple-helical structure. Chem. Phys. Lett. 559, 88–93. 10.1016/j.cplett.2012.12.061

[B40] Rupp M. Walter N. Lau E. Worlicek M. Kurtz S. M. Alt V. (2021). Recent trends in revision knee arthroplasty in Germany. Sci. Rep. 11 (1), 15479. 10.1038/s41598-021-94988-7 34326421 PMC8322047

[B41] Schäfer P. Fink B. Sandow D. Margull A. Berger I. Frommelt L. (2008). Prolonged bacterial culture to identify late periprosthetic joint infection: a promising strategy. Clin. Infect. Dis. 47 (11), 1403–1409. 10.1086/592973 18937579

[B42] Termaat M. F. (2005). The accuracy of diagnostic imaging for the assessment of chronic osteomyelitis: a systematic review and meta-analysis. J. Bone Jt. Surg. Am. 87 (11), 2464–2471. 10.2106/jbjs.d.02691 16264122

[B43] Tübel J. Maier E. Jegen M. Marthen C. Obermeier A. Haug A. T. (2021). Patient-specific effects of soluble factors from Staphylococcus aureus and Staphylococcus epidermidis biofilms on osteogenic differentiation of primary human osteoblasts. Sci. Rep. 11 (1), 17282. 10.1038/s41598-021-96719-4 34446785 PMC8390505

[B44] Unkila-Kallio L. Kallio M. J. Peltola H. Eskola J. (1994). Serum C-reactive protein, erythrocyte sedimentation rate, and white blood cell count in acute hematogenous osteomyelitis of children. Pediatrics 93 (1), 59–62. 10.1542/peds.93.1.59 8265325

[B45] Varma S. Orgel J. P. Schieber J. D. (2016). Nanomechanics of type I collagen. Biophys. J. 111 (1), 50–56. 10.1016/j.bpj.2016.05.038 27410733 PMC4945622

[B46] Vastel L. Meunier A. Siney H. Sedel L. Courpied J. P. (2004). Effect of different sterilization processing methods on the mechanical properties of human cancellous bone allografts. Biomaterials 25 (11), 2105–2110. 10.1016/j.biomaterials.2003.08.067 14741625

[B47] Vieira A. L. Nespeca M. G. Pavini W. D. Ferreira E. C. Gomes Neto J. A. (2019). A user-friendly excel spreadsheet for dealing with spectroscopic and chromatographic data. Chemom. Intelligent Laboratory Syst. 194, 103816. 10.1016/j.chemolab.2019.103816

[B48] Walter N. Rupp M. Lang S. Alt V. (2021a). The epidemiology of fracture-related infections in Germany. Sci. Rep. 11 (1), 10443. 10.1038/s41598-021-90008-w 34001973 PMC8128870

[B49] Walter N. Rupp M. Hinterberger T. Alt V. (2021b). Protheseninfektionen und die zunehmende Bedeutung psychologischer Komorbiditäten. Der Orthop. 50 (10), 859–865. 10.1007/s00132-021-04088-7 PMC7942820 33751197

[B50] Wolff J. (1893). Das gesetz der transformation der knochen. DMW-Deutsche Med. Wochenschr. 19 (47), 1222–1224. 10.1055/s-0028-1144106

[B51] Wright J. A. Nair S. P. (2010). Interaction of staphylococci with bone. Int. J. Med. Microbiol. 300 (2-3), 193–204. 10.1016/j.ijmm.2009.10.003 19889575 PMC2814006

